# Association Between Sleep Patterns and the Risk of Cardiometabolic Diseases or Cardiometabolic Multimorbidity and the Mediating Role of Metabolic Biomarkers: A Prospective Study of the UK Biobank Cohort

**DOI:** 10.3390/healthcare14152296

**Published:** 2026-07-29

**Authors:** Duqiu Liu, Yi Guo, Guochen Li, Chenxing Yang, Jingjie Xiong, Shan Deng, Ru Chen

**Affiliations:** 1 Cardiovascular Center, Liyuan Hospital, Tongji Medical College, Huazhong University of Science and Technology, Wuhan 430060, China; d202182136@hust.edu.cn (D.L.); d202282268@hust.edu.cn (Y.G.); d202382048@hust.edu.cn (J.X.); 2 Clinic Center of Human Gene Research, Union Hospital, Tongji Medical College, Huazhong University of Science and Technology, Wuhan 430010, China; 3 Department of Cardiology, The Fifth Affiliated Hospital of Southern Medical University, Guangzhou 510900, China; 4 Department of Epidemiology, NHC Key Laboratory for Health Technology Assessment, Fudan University School of Public Health, Shanghai 200032, China; liguochen1225@fudan.edu.cn; 5 Department of Epidemiology and Biostatistics, School of Public Health, Tongji Medical College, Huazhong University of Science and Technology, Wuhan 430030, China; bmohufbmpi@163.com; 6 Department of Cardiology, Union Hospital, Tongji Medical College, Huazhong University of Science and Technology, Jie Fang Road No. 1277, Wuhan 430022, China; 7 Hubei Key Laboratory of Metabolic Abnormalities and Vascular Aging, Huazhong University of Science and Technology, Wuhan 430022, China; 8 Hubei Clinical Research Center of Metabolic and Cardiovascular Disease, Huazhong University of Science and Technology, Wuhan 430022, China

**Keywords:** sleep pattern, metabolite, cardiometabolic disease, cardiometabolic multimorbidity, UK biobank

## Abstract

**Aim:** To identify the relationship between joint sleep patterns and the incidence of cardiometabolic diseases (CMDs) and multimorbidity (CMM) as well as the mediating effects of plasma metabolites. **Methods:** This prospective study included 190,827 participants from the UK Biobank. The joint sleep pattern was evaluated by the healthy sleep score according to five sleep factors (chronotype, sleep duration, insomnia, snoring, and excessive daytime sleepiness). Cox proportional hazards models were utilized to evaluate the relationship between sleep-related metabolites and the incidence of CMM and CMDs. Mediation analyses were conducted to quantify the potential mediating effects of circulating metabolites. **Results:** After a median follow-up of 13.5 years, 24,602 incident cases of CMDs and 2740 incident cases of CMM were identified. Per 1-decrement of sleep score was associated with 10.5% and 15.1% increased risks of CMDs and CMM, respectively. However, sleep was not associated with the progression from the first disease to CMM. A total of 152 metabolites were identified to be associated with sleep patterns, among which lipoproteins emerged as the predominant class. Most of the metabolites showed mediation effects on the association between sleep and outcomes. **Conclusions:** Poor sleep pattern was associated with increased risk of CMDs and CMM. Differences in metabolites, especially lipoproteins, might partially explain the association between sleep pattern and cardiometabolic health.

## 1. Introduction

Cardiometabolic diseases (CMDs), mainly including coronary heart disease (CHD), stroke, and type 2 diabetes, represent the leading cause of morbidity and mortality worldwide [1,2,3]. Approximately 254.3 million cases of CHD and 93.8 million cases of stroke were reported worldwide in 2021 [4,5], and an estimated 828 million adults were reported to be living with diabetes by 2022, with age-standardized prevalence rates of 13.9% in women and 14.3% in men [6]. As the global population continues to grow and age, the prevalence and mortality rates attributed to CMDs are anticipated to rise, potentially imposing significant socioeconomic burdens and reducing life expectancy. Moreover, the co-occurrence of multiple CMDs significantly complicates clinical management and elevates the risk of adverse health outcomes [2]. Thus, it is imperative to explore novel modifiable risk factors and the underlying mechanisms linking these factors to CMDs and cardiometabolic multimorbidity (CMM).

Sleep is increasingly recognized as a crucial factor influencing cardiometabolic health [7,8]. It is estimated that over 30% of the global population suffers from sleep disorders [9,10]. Sleep-related factors such as duration, insomnia, snoring, chronotype, and excessive daytime sleepiness have been linked to CMDs, including CHD, T2DM, stroke, obesity, dyslipidemia, and hypertension [7,11,12,13,14,15]. Notably, individuals who maintain consistently favorable sleep patterns demonstrate a significantly lower risk of CHD and stroke, independent of their genetic predispositions [15]. Concerning CMM, a dose–response relationship between sleep duration and CMM has been observed among older adults in China [16]. Additionally, night shift work has been associated with an elevated risk of CMM in hypertensive patients [17]. Evidence suggests that higher or improved healthy sleep scores over time are correlated with a reduced risk of CHD and stroke [18,19]. Moreover, a healthy sleep pattern has been associated with a lower risk of all-cause mortality in individuals with CMM, as well as a decreased likelihood of adverse outcomes among those with hypertension [17,20]. Despite these findings, prospective studies with large sample sizes that examine the relationship between joint sleep patterns and the incidence of CMDs, especially CMM, are still lacking.

Metabolomics has emerged as an invaluable tool for identifying biomarkers and unraveling the potential mechanisms underlying various diseases [21]. Lipid metabolites have been reported to be associated with the consumption of coffee, tea, and caffeine, as well as with the incidence of CMM [22]. A study identified 121 metabolites that demonstrated significant mediation effects between preserved ratio impaired spirometry and the development of T2DM [23]. Furthermore, a metabolic signature linked to a healthy lifestyle explained 20% of the correlation between healthy lifestyle scores and cardiovascular disease [24]. While several studies have reported on the mediating effects of metabolites in the relationship between sleep disorders and cardiometabolic health [25,26,27], these studies typically involved small sample sizes and did not evaluate the metabolic signatures associated with combined sleep patterns. Therefore, metabolites are likely to play a significant mediating role in the pathway from sleep disorders to the incident CMDs. However, the metabolic signatures associated with joint sleep patterns and the mediating role of metabolic signatures between sleep patterns and CMDs, as well as CMM, remain largely unexplored.

To fill the gaps in the existing literature, we aimed to identify the metabolic profile related to joint sleep patterns and to explore the mediating effects of this metabolic signature in the relationship between sleep patterns and incident CMDs as well as CMM.

## 2. Materials and Methods

### 2.1. Study Population

Participants in this study were enrolled from the UK Biobank, a prospective cohort that recruited over 500,000 individuals aged 40–69 from 22 assessment centers across the UK between 2006 and 2010 [28]. Ethical approval for the UK Biobank was granted by the North West Multi-Centre Research Ethics Committee (21/NW/0157). Each participant provided electronically signed consent at the time of their initial enrollment. Baseline characteristics of each participant, including demographic, lifestyle, reproductive, environmental, genetic, medical, physical, and biochemical data, were assessed through a self-administered touchscreen questionnaire, physical and functional measurements, and computer-assisted interviews.

The data utilized in this study were extracted under access application number 109546. Initially, 502,250 individuals were included in the study. Participants without complete data on sleep patterns (n = 90,761), and those with a history of T2DM, stroke, or CHD at baseline (n = 45,722), were excluded. Additionally, 96 participants who withdrew their informed consent during the course of the study, as well as 174,884 participants lacking nuclear magnetic resonance (NMR) data on metabolites, were excluded. Finally, a total of 190,827 participants were included in the analysis. A flow diagram depicting the recruitment process is provided in Figure S1.

### 2.2. Assessment of Sleep Patterns

The sleep behaviors assessed in this study encompassed sleep duration, chronotype, insomnia, snoring, and daytime sleepiness. All the behaviors were self-reported by participants via touchscreen questionnaires. A sleep score was calculated based on the five sleep behaviors following a previous study [19]. Sleep durations of 7–8 h per day, a “morning” or “more morning than evening” chronotype, absence of insomnia symptoms, no self-reported snoring, and “never/rarely” or “sometimes” daytime sleepiness were defined as low-risk sleep factors for each behavior. High-risk factors were assigned a score of 0, while low-risk factors received a score of 1. The total sleep score, ranging from 0 to 5, was the sum of the scores from all five behaviors. The overall sleep pattern was further classified as healthy (score ≥ 4), intermediate (2 ≤ score ≤ 3), or poor (score ≤ 1) based on the total sleep score.

### 2.3. Outcomes

The primary outcome was the incidence of total CMDs and CMM. The secondary outcomes included the incidence of CHD, stroke, and T2DM. These outcomes were defined using the tenth edition of the International Classification of Diseases (ICD-10) codes and an algorithm developed by the UK Biobank (Table S1). CMDs were defined by the presence of either CHD, stroke or T2DM. CMM refers to the presence of two distinct cardiometabolic disorders within the same individual, which may include any combination of T2DM, stroke, and CHD. The onset of CMM was defined as the date of diagnosis of the second cardiometabolic disease. Outcome information was identified through linkage to self-reported medical conditions, primary care, inpatient hospital data, and death registries. Detailed information regarding the linkage procedure is available online (https://biobank.ndph.ox.ac.uk/showcase/refer.cgi?id=115559, accessed on 31 October 2022 for England, 31 August 2022 for Scotland, and 31 May 2022 for Wales for hospital inpatient data, and 30 November 2022 for death data).

### 2.4. NMR Metabolomics

Metabolic biomarkers were assessed from randomly selected EDTA plasma samples (aliquot 3) using a high-throughput NMR-based metabolic profiling platform developed by Nightingale Health Ltd. The measurements were conducted between June 2019 and April 2020 (Phase 1) and from April 2020 to June 2022 (Phase 2), utilizing eight spectrometers at Nightingale Health, based in Finland. Detailed information regarding the profiling platform and experimental protocols can be found on the UK Biobank website (https://biobank.ndph.ox.ac.uk/ukb/refer.cgi?id=130, accessed on 31 October 2022). A total of 251 metabolic biomarkers were quantified in molar concentration units. Of these biomarkers, 168, including lipids, amino acids, ketones, and glycolytic metabolites, were measured in absolute levels, and this study primarily focused on the 168 directly measured metabolites. Lipoprotein subclass analyses assessed lipid concentrations and compositions—triglycerides, phospholipids, total cholesterol, cholesterol esters, free cholesterol, and total lipid content within each subclass.

### 2.5. Covariates

Covariates included in the current study were obtained from the baseline survey, involving age, sex, ethnicity, socioeconomic status, lifestyle, educational attainment, household income, common disease, family history and information on drug use. Townsend deprivation index, incorporating unemployment, non-car ownership, non-home ownership, and household overcrowding, was calculated to evaluate socioeconomic status [29]. BMI was calculated as weight in kilograms divided by the square of height in meters, with measurements taken by a trained nurse during the initial assessment. Smoking status, alcohol consumption, and physical activity were self-reported at baseline through verbal interviews and touchscreen questionnaires. Smoking and alcohol consumption were categorized as never, past, or current. Physical activity was assessed by the total metabolic equivalent of task (MET) minutes per week for all activities. The high physical activity group comprised those performing >150 min/week of moderate activity and/or >75 min/week of vigorous activity. The moderate group included individuals with moderate activity < 150 min/week and vigorous activity < 75 min/week. The low group consisted of participants with no documented moderate or vigorous physical activity. Hypertension was defined as either a self-reported history of hypertension or a measured blood pressure of ≥140/90 mmHg at baseline. A family history of diabetes or cardiovascular disease (CVD) was determined based on self-reported parental history.

### 2.6. Statistical Analysis

Continuous variables were presented as median (interquartile ranges), while categorical variables were displayed as numbers (percentages). Kruskal–Wallis tests (for continuous variables) and Chi-squared tests (for categorical variables) were employed to assess differences in baseline characteristics. Follow-up time was calculated from the baseline date to the diagnosis of the outcome, death, or censoring date, whichever occurred first. Cox proportional hazards models were used to examine the relationship between sleep patterns and the risk of incident CMDs as well as CMM, with results expressed as hazard ratios (HRs) and 95% confidence intervals (CIs). The proportional hazards assumption was tested using the Schoenfeld residuals method. The model was adjusted for age, sex, ethnicity, Townsend deprivation index, educational attainment, annual household income, alcohol intake, physical activity, smoking status, BMI, hypertension, family history of diabetes, and family history of cardiovascular disease. Participants with missing data on covariates (Table S2) were excluded from the analysis.

Next, we employed multi-state models to assess the dynamic associations of sleep patterns with transitions between cardiometabolic disease states by using the mstate R package 0.3.3 [30]. Two transition patterns were defined. The primary model (Pattern A) included five transitions: baseline to first cardiometabolic disease (FCMD), baseline to death, FCMD to CMM, FCMD to death, and CMM to death. A secondary analysis (Pattern B) examined transitions from baseline to specific first CMDs, each CMD to CMM, and CMD to death.

To identify metabolites associated with sleep patterns (per 1 decrement), multiple linear regression models were employed. Metabolites with a false discovery rate (FDR)-adjusted *p* < 0.05 were deemed statistically significant and selected as candidates. Subsequently, Cox proportional hazards models were utilized to evaluate the relationship between these sleep pattern-related metabolites and the incidence of CMM, as well as subtypes of CMDs, including CHD, stroke, and T2DM. We next investigated the mediating role of the selected metabolites in the relationship between sleep patterns and the incidence of CMM and subtypes of CMDs. Only metabolites that showed significant associations with both the sleep pattern and the clinical outcomes were advanced to formal mediation analysis. The “mediation” package in R software was utilized to estimate mediation proportions, with confidence intervals (CIs) calculated using a quasi-Bayesian Monte Carlo method based on 2000 simulations. We assumed that the total effect could be decomposed into two components: the natural direct effect (NDE, representing the direct pathway: sleep patterns → CMM/CMD) and the natural indirect effect (NIE, representing the mediated pathway: sleep patterns → metabolites → CMM/CMD) (Figure S2).

Several sensitivity analyses were conducted to evaluate the robustness of the findings. First, we excluded participants who developed CMDs within the first two years of follow-up. Second, to avoid the potential influence of medications on plasma lipids, we excluded participants who were using lipid-lowering medications at baseline. Third, to account for the varying contributions of individual sleep components to the outcomes, we constructed a weighted sleep score using the formula: weighted sleep score = (β_1_ × factor_1_ + β_2_ × factor_2_ + … + β_5_ × factor_5_) × (5/Σβ) [19]. Finally, to mitigate any potential influence of the sleep–adiposity–dyslipidemia pathway, we performed an additional sensitivity analysis in which BMI was removed from the covariates.

All analyses were performed using SAS 9.4 (SAS Institute, Cary, NC, USA) and R 4.3.1. A two-sided *p* < 0.05 was considered statistically significant.

## 3. Results

### 3.1. Baseline Characteristics of the Participants

The baseline characteristics of the 190,827 participants included in the final analysis are presented in Table 1. Among the participants, 73,129 were found to have healthy sleep patterns, while 109,933 exhibited intermediate sleep patterns, and 7765 showed poor sleep patterns. People with poorer sleep pattern tend to be younger and had higher proportion of males (all *p* < 0.0001). Meanwhile, individuals with poor and intermediate sleep pattern were more likely to be deprived and less-educated, and had lower annual household income (all *p* < 0.0001). Those with poor and intermediate sleep patterns had lower physical activity and higher BMI (all *p* < 0.0001). Alcohol intake and smoking were more common in individuals with poor and intermediated sleep pattern (all *p* < 0.0001). In addition, people with poor and intermediate sleep patterns were more likely to have hypertension and family history of diabetes. Although individuals with healthy sleep patterns were more likely to have family history of cardiovascular disease, they were less likely to use lipid-lowering drugs (all *p* < 0.0001).

### 3.2. Association Between Sleep Pattern and Risk of CMDs

After a median follow-up of 13.5 (12.6–14.2) years, 24,602 incident cases of CMDs were observed, including 8203 cases of T2DM, 14,683 cases of CHD, and 4594 cases of stroke (Table 2). In addition, 2740 cases of incident CMM were noted. Compared to participants with healthy sleep pattern, people with intermediate and poor sleep pattern were associated with 20.1% (HR 1.201, 95% CI 1.103–1.308) and 65.3% (HR 1.653, 95% CI 1.419–1.926) increased risk of CMM after multivariable adjustment. Additionally, intermediate (HR 1.148, 95 CI% 1.117–1.180) and poor sleep pattern (HR 1.430, 95 CI% 1.352–1.512) were associated with increased risk of total CMDs. Per 1-decrement of sleep score was associated with 15.1% (HR 1.151, 95% CI 1.108–1.196) and 10.5% (HR 1.105, 95% CI 1.090–1.119) increased risks of CMM and total CMDs. For each subtype of CMDs, poor sleep pattern was also related with increased risk of all three CMDs subtypes, with HR (95% CI) 1.613 (1.479–1.758), 1.240 (1.078–1.427) and 1.371 (1.272–1.477) for T2DM, stroke and CHD, respectively. Per 1 decrement of sleep score was associated with 14.3%, 6.0% and 10.1% increased risk of T2DM, stroke and CHD.

In the multi-state model, per 1-decrement of healthy sleep score was associated with 10.44% and 5.26% increased risks of transition from baseline to FCMD and death (Table S3). Consistently, per 1 decrement of healthy sleep score was associated with increased risks of transition from baseline to T2DM, stroke and CHD. However, the decreased healthy sleep score was not associated with progression to CMM from either FCMD or specific CMDs.

### 3.3. Association Between Sleep Pattern-Related Metabolites and Risk of CMM as Well as CMDs

We subsequently examined the metabolic profiles associated with sleep patterns. A total of 152 metabolites (FDR < 0.05) were identified as significantly associated with sleep patterns (Table S4). The top three metabolites most strongly correlated with the sleep score (per 1 decrement) included monounsaturated fatty acids, glycoprotein acetyls, and triglycerides in medium low-density lipoprotein (LDL), with standardized regression coefficients (95% CIs) of 0.049 (0.047–0.051), 0.047 (0.045–0.049), and 0.046 (0.043–0.048), respectively.

**Figure 1:**
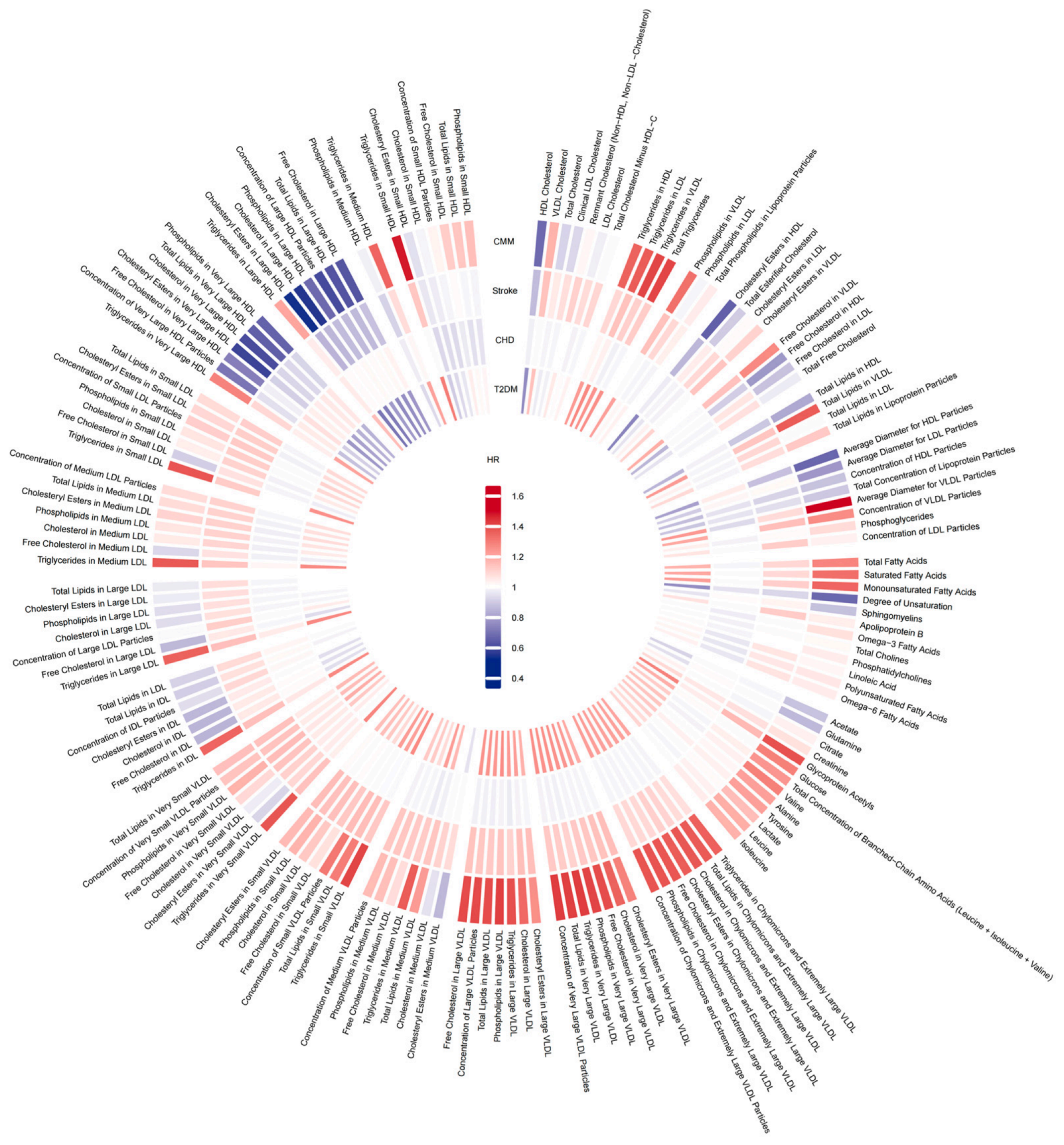
Associations between sleep score-related metabolic biomarkers with incident CMDs and CMM. Circular heatmap showing the associations between healthy sleep score-related metabolic biomarkers with the risks of T2DM, stroke, CHD and CMM. Metabolites are displayed as segments around the chart, with the four outcomes shown from the inner to outer rings. Color intensity represents the magnitude of hazard ratios (HRs), with darker shades indicating larger effect sizes and lighter shades indicating HRs closer to 1.0. Red and blue denote positive and negative associations, respectively. All associations were derived from Cox proportional hazard models adjusted for age, sex, ethnicity, Townsend deprivation index, education attainment, annual household income, alcohol intake, physical activity, smoking status, body mass index, hypertension, history of diabetes, and cardiovascular disease. Abbreviations: CHD, coronary heart disease; CMM, cardiometabolic multimorbidity; HR, hazard ratio; T2DM, type 2 diabetes mellitus.

**Figure 2:**
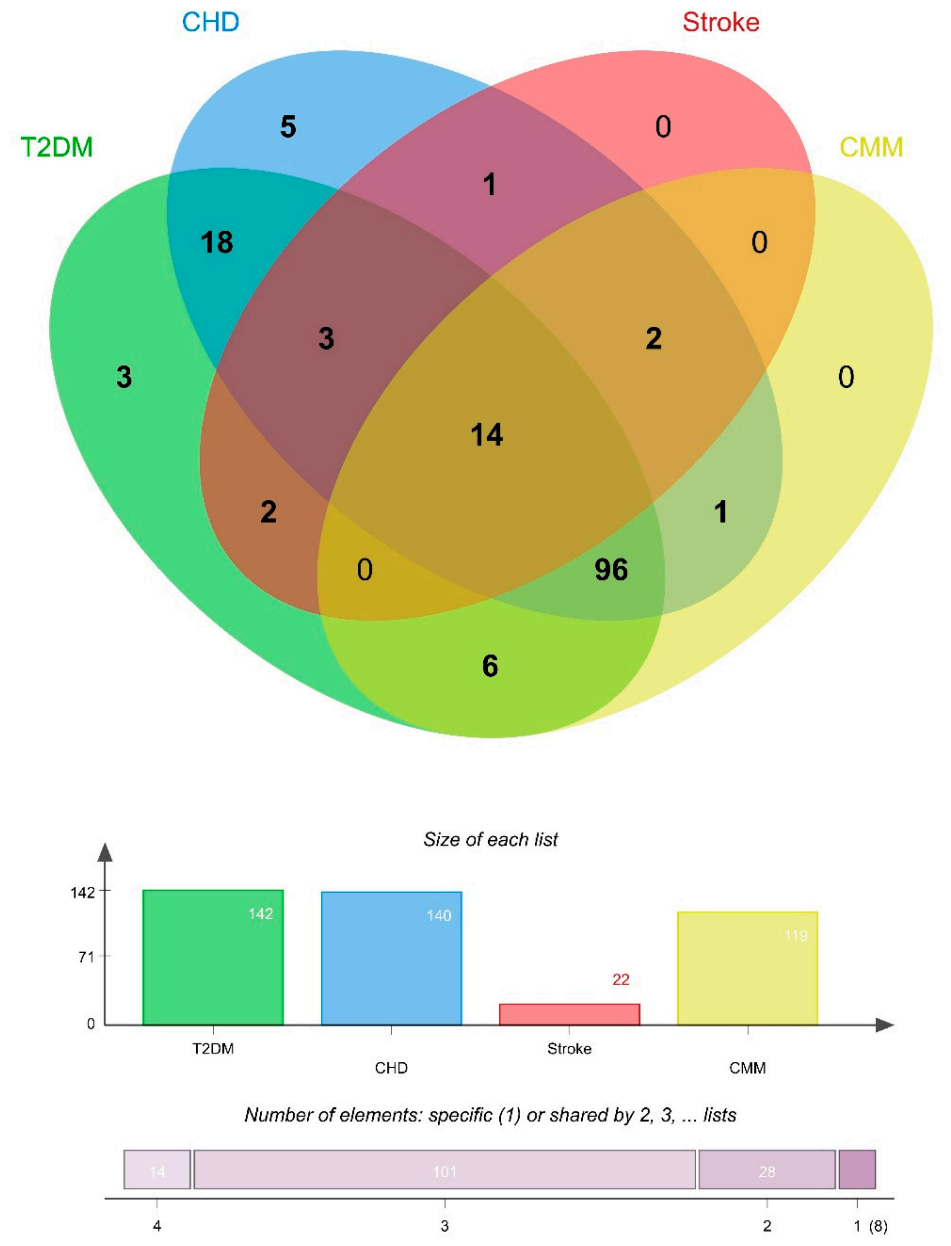
Number of metabolites associated with CMDs and the overlaps among them. Abbreviations: CHD, coronary heart disease; CMM, cardiometabolic multimorbidity; T2DM, type 2 diabetes mellitus.

Among the 152 metabolites related to sleep pattern, 119, 142, 140, and 22 were significantly associated with the risk of CMM, T2DM, CHD, and stroke, respectively (*FDR* < 0.05) (Figure 1 and Table S5). A total of 143 metabolites were shared by at least 2 outcomes, and 14 metabolites were shared by all four outcomes (Figure 2). Among the 14 metabolites, the majority (N = 9) belonged to lipoprotein subclasses (Table S6). Other common metabolites included total concentration of lipoprotein particles, concentration of HDL particles, average diameter for VLDL particles, degree of unsaturation and creatinine.

### 3.4. Mediation Effects of Metabolites in the Association Between Sleep Patterns and CMM as Well as CMDs

**Figure 3:**
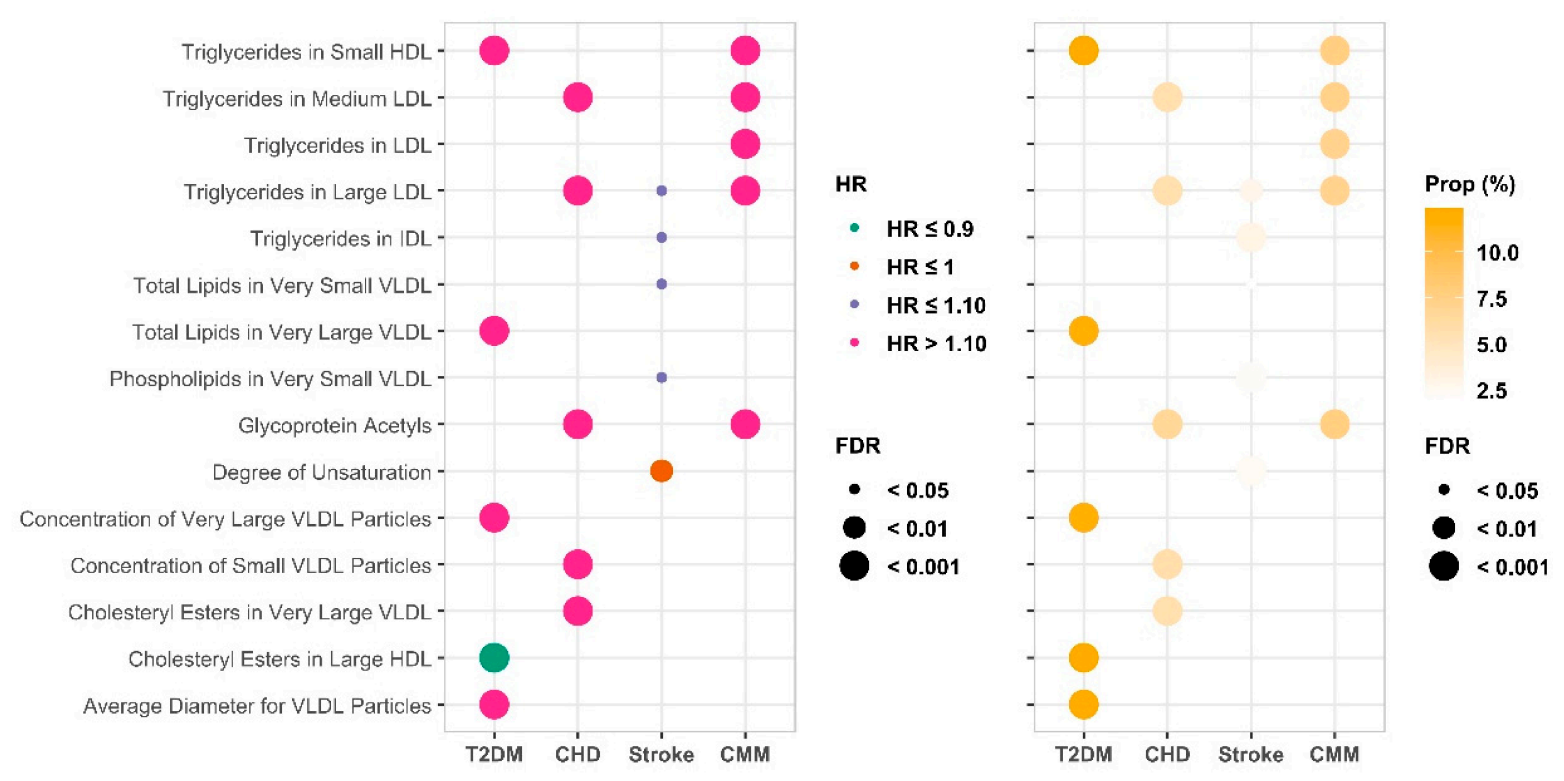
Associations and mediation effects of sleep score-related metabolic biomarkers with incident CMDs and CMM. Models adjusted for age, sex, ethnicity, Townsend deprivation index, education attainment, annual household income, alcohol intake, physical activity, smoking status, body mass index, hypertension, history of diabetes, and cardiovascular disease. Abbreviations: CHD, coronary heart disease; CMM, cardiometabolic multimorbidity; HDL, high-density lipoprotein; HR, hazard ratio; IDL, intermediate-density lipoprotein; LDL, low-density lipoprotein; T2DM, type 2 diabetes mellitus; VLDL, very low-density lipoprotein.

We next assessed the mediation effects of metabolites associated with both sleep patterns and outcomes (Figure 3 & Table S7). For CMM, all 119 metabolites exhibited significant mediation effects (*FDR* < 0.05). The top five mediators were glycoprotein acetyls, triglycerides in small HDL, triglycerides in medium LDL, triglycerides in LDL, and triglycerides in large LDL, with mediation proportions (95% CIs) of 7.68% (5.81–11.13%), 7.68% (5.89–10.95%), 7.36% (5.48–11.07%), 7.12% (5.31–9.72%), and 7.10% (5.26–9.87%), respectively. Furthermore, triglycerides in all other subclasses of lipoproteins displayed significant mediation effects.

For T2DM, all 142 metabolites exhibited mediation effects, with the top five mediators being cholesteryl esters in large HDL (mediation proportion 12.33%, 95% CI 9.90–15.13%), triglycerides in small HDL (mediation proportion 12.26%, 95% CI 10.24–15.37%), average diameter for VLDL particles (mediation proportion 12.15%, 95% CI 10.24–14.68%), concentration of very large VLDL particles (mediation proportion 11.72%, 95% CI 9.88–14.70%), and total lipids in very large VLDL (mediation proportion 11.71%, 95% CI 9.61–14.62%). For CHD, all 140 metabolites displayed mediation effects. The top five mediators were glycoprotein acetyls, concentration of small VLDL particles, triglycerides in large LDL, cholesteryl esters in very large VLDL, and triglycerides in medium LDL, with mediation proportions (95% CIs) of 6.67% (5.30–8.19%), 5.79% (4.66–7.31%), 5.69% (4.54–7.23%), 5.66% (4.39–7.22%), and 5.62% (4.58–7.11%), respectively. Regarding the relationship between sleep pattern and stroke, 22 metabolites demonstrated mediation effects. The top five mediators were triglycerides in IDL, triglycerides in large LDL, degree of unsaturation, phospholipids in very small VLDL, and total lipids in very small VLDL, with mediation proportions (95% CIs) of 3.07% (1.12–7.24%), 2.87% (0.76–7.35%), 2.45% (1.02–6.57%), 2.25% (0.77–6.11%), and 1.98% (0.52–4.28%), respectively.

### 3.5. Sensitivity Analysis

The results remained robust across a series of sensitivity analyses. Excluding participants with incident CMDs within the first two years of follow-up, as well as those taking lipid-lowering medications at baseline, yielded consistent results (Table S8). When the weighted sleep score was used in place of the unweighted score, the results were consistent with those of the primary analysis (Table S9). Additionally, omitting BMI from the covariates did not appreciably affect the associations (Table S10).

## 4. Discussion

In this prospective cohort study including 190,827 UK adults, we observed a significant association between poorer sleep patterns and heightened risks of CMDs and CMM. Notably, poorer sleep did not appear to drive the transition from an initial CMD diagnosis to CMM. Furthermore, sleep pattern was associated with 152 metabolites across various metabolic pathways, mainly involving lipoproteins, fatty acids, amino acids and glycolysis. In total, 119 of the metabolites were associated with incident CMM, while 142, 140, and 22 of the metabolites were related with T2DM, CHD and stroke, respectively. Most of the metabolites showed mediation effect in the relationship between sleep pattern and CMDs, with lipoproteins representing the predominant class.

By utilizing the large prospective cohort with long-term follow-up from the UK Biobank, we observed that a poor joint sleep pattern was significantly associated with an elevated risk of incident CMM and CMDs. In hypertensive patients, a healthy sleep pattern was associated with lower risks of CMM and CMD subtypes (CHD, T2DM, stroke), aligning with our findings in the general population [31]. A cross-sectional study identified that a night sleep duration of less than 5 h ranked as one of the top two risk factors associated with CMM in women [32]. A sleep duration of less than 5 h was related to an increased risk of incident CMM [33]. Obstructive sleep apnea (OSA) was shown to significantly increase the occurrence of major adverse cardiovascular and cerebrovascular events in patients with CMM [34]. However, these studies did not assess the relationship between joint sleep patterns and CMM. Both the weighted and unweighted joint sleep scores showed significant associations with the risks of CMDs and CMM, indicating that comprehensive sleep management may play a crucial role in cardiometabolic health.

The influence of sleep on the progression from baseline health to FCMD and onward to CMM or death was previously unclear. Our findings indicated that a lower healthy sleep score significantly increases the risk of progressing from health to FCMD and elevates the risk of death. However, it does not significantly influence progression from established FCMD to CMM or death. Notably, sleep patterns in participants with existing CMDs were associated with worse clinical outcomes, suggesting that sleep retains prognostic importance even after disease onset [35,36]. One possible explanation is that after the onset of a cardiometabolic condition, patients might adopt healthier lifestyles (including a shift toward healthier sleep patterns) or initiate pharmacological treatments, which may attenuate the influence of baseline sleep on subsequent disease progression. Taken together, these findings indicate that the protective role of healthy sleep primarily operates through primary prevention, reducing the risk of developing the first cardiometabolic disease, whereas progression to multimorbidity may be governed by additional clinical and behavioral factors beyond baseline sleep. Future studies with repeated sleep assessments and extended follow-up are needed to clarify the role of sleep in disease progression.

Our study is among the first to identify metabolites associated with joint sleep patterns in a large prospective cohort. We discovered that multiple metabolites, particularly lipid species, were influenced by sleep patterns. Previous research has noted alterations in plasma lipids during poor sleep, which is consistent with our findings [26,37,38]. However, most prior studies were limited in size and focused on isolated sleep behaviors. For instance, a cross-sectional study identified a few metabolites within specific biochemical pathways associated with longer sleep duration and sleep variability [39]. A small randomized controlled trial found that recurrent sleep restriction impacted various metabolic pathways, particularly amino acids, in adults at risk for T2DM [40]. Other studies have explored how misaligned sleep/wake cycles disrupt the rhythms of circulating metabolites [41], and patients with OSA have demonstrated altered plasma metabolite profiles [42]. A study combining blood transcriptome and metabolome data found changes in cholesterol and inflammatory pathways in response to both experimental and habitual sleep restriction [43]. Our study filled gaps in the previous literature by offering a more comprehensive understanding of how joint sleep patterns influence metabolic changes and revealed abundant metabolites. However, since the serum sample and sleep patterns were collected both as baseline, additional randomized controlled trials and longitudinal studies are necessary to clarify the impact of different sleep patterns on plasma metabolites and the subsequent shifts following modifications to sleep habits.

Mechanisms linking poor sleep health with CMDs are not fully understood. The prevailing view is that sleep behaviors may influence cardiometabolic health in part through metabolic regulation [44]. Insufficient sleep has been associated with weight gain and elevated blood pressure, which may in turn adversely affect cardiometabolic health [45]. Sleep deprivation disrupts glucose metabolism, insulin resistance, inflammation, oxidative stress, and autonomic nervous system function, all of which contribute to worsened cardiovascular health [11,46,47,48,49]. Serum metabolomic analysis may serve as a useful tool to explore metabolic alterations associated with both sleep patterns and CMDs, generating hypotheses about potential mediating pathways. A previous study found that prolonged sleep deprivation altered inflammatory and cholesterol pathways at the gene expression level and serum lipoproteins, suggesting a potential link to increased CMD risk [43]. Experimental studies on sleep-restricted mice showed significant changes in metabolites such as lipoproteins and triglycerides, with all fatty acid components decreasing after sleep loss [50]. In this study, we identified 152 metabolites associated with poor sleep patterns, of which 119 were linked to the incident risk of CMM. Additionally, 142, 140, and 22 metabolites related to sleep patterns were associated with the risks of T2DM, CHD, and stroke, respectively. These metabolites span components of lipids, fatty acids, amino acids, glycolysis, and fluid balance, highlighting the potential relevance of sleep patterns to metabolic regulation. Moreover, most identified metabolites were associated with both sleep patterns and CMD risk and showed mediating effects, supporting the hypothesis that metabolic disturbances may mediate the sleep–cardiometabolic link. However, given the cross-sectional design of our sleep–metabolite analysis, these results are exploratory and hypothesis-generating rather than conclusive of causal mediation. Future experimental studies and longitudinal cohort studies with repeated measurements are warranted to validate the causal role of these metabolites and to elucidate the underlying biological mechanisms.

Among the sleep pattern-related metabolites, abundant lipid metabolites, particularly HDL, LDL, and VLDL, were associated with the risk of CMM. Four of the top five mediators between poor sleep patterns and CMM were lipoprotein-associated triglycerides. This finding appears to contrast with the conventional emphasis on cholesterol over triglycerides as a therapeutic target in CMD management. However, given that serum samples were collected under non-fasting conditions, measurement bias in triglyceride levels cannot be ruled out. Additionally, glycoprotein acetyls, a proteoglycan inflammatory biomarker, ranked top among the mediators, supporting the link between metabolic disorders and inflammation [51]. Several metabolites related to amino acids, glycolysis, and fluid balance also showed mediating effects. Creatinine exhibited a positive association with incident CMM, CHD, and stroke, underscoring its potential utility in clinical risk assessments of chronic metabolic diseases. Collectively, by evaluating the associations of metabolites across multiple pathways with both sleep patterns and disease outcomes, our findings provide exploratory insights into the metabolic profiles that accompany poor sleep and that may be relevant to the development of CMM and CMDs. These observations generate hypotheses for future prospective and experimental studies to examine whether these metabolites play a causal mediating role.

Interestingly, we identified only 22 sleep-pattern-related metabolites associated with the risk of stroke. Several metabolites common to CMM, CHD, and T2DM were not shared with stroke, which corroborates previous findings that stroke shares relatively few metabolites with CHD and T2DM [52]. One possible explanation for this disparity is that the blood–brain barrier may hinder the entry of metabolites. The lipoproteins linked to stroke were primarily components within very small VLDL and small HDL, lending support to this hypothesis. Free fatty acids, however, are capable of crossing the blood–brain barrier [53]. In our study, linoleic acid, omega-6 fatty acids and polyunsaturated fatty acids were negatively associated with incident stroke. The health effects of polyunsaturated fatty acids in stroke remain controversial. α-linolenic acid has been linked to a reduced risk of stroke [54]. Yet, a large genome-wide association study of circulating polyunsaturated fatty acids and Mendelian randomization analyses did not affirm a protective role of circulating polyunsaturated fatty acids against stroke risk [55]. Additionally, there is no established benefit of omega-3 polyunsaturated fatty acid supplementation for the primary or secondary prevention of stroke [56]. These discrepancies may stem from the complex subtypes of polyunsaturated fatty acids, warranting further research into the effects of specific types on stroke.

Still, this study has several limitations. First, all sleep behaviors were self-reported at baseline, which could introduce potential bias, and further studies with objective methods evaluating sleep quality should be considered. Second, plasma metabolomics were measured only once at baseline using the NMR profiling platform. The fundamental biological pathways linking sleep regulation to lipoprotein metabolism are largely governed by conserved circadian and metabolic mechanisms, suggesting that the core associations are likely to remain valid over time. However, the alteration of metabolomic profile in a long-term duration was unknown and many other circulating metabolites remain to be complemented through utilizing mass spectrometry-based platforms. And the possibility of reverse causality should be acknowledged. Third, plasma samples were not fasting, which may increase the variation in metabolite concentration within and between individuals. Fourth, the majority of our participants were white. Given that sleep behaviors, metabolic phenotypes, and cardiometabolic risk are shaped by population-specific determinants—including genetic architecture, nutritional patterns, lifestyle behaviors, social determinants, and health system factors—the extrapolation of our results to diverse ethnic populations warrants caution. Fifth, though we adjusted for multiple variables, residual and unknown confounding such as diet quality, sleep apnea severity, mental health status, and occupational schedules could not be entirely excluded. Sixth, secular changes in lifestyle factors—particularly increased screen time, altered light exposure patterns, and the heightened prevalence of mental health issues following the COVID-19 pandemic—may influence the magnitude of these associations in contemporary populations.

## 5. Conclusions

Poor sleep patterns were associated with an increased risk of incident CMDs but did not predict progression to CMM. Lipoprotein-related metabolites emerged as potential mediators between sleep health and CMDs, and longitudinal and experimental studies are warranted to confirm these mechanistic pathways.

## Figures and Tables

**Table 1:** Baseline characteristics of the study participants according to sleep pattern and healthy sleep score.

Characteristics	Sleep Pattern/Healthy Sleep Score			*p*-Values
	Healthy Sleep (Score ≥ 4)	Intermediate Sleep (2 ≤ Score ≤ 3)	Poor Sleep (Score ≤ 1)	
Count (%)	73,129	109,933	7765	
Sex of male (%)	29,828 (40.79)	49,055 (44.62)	3928 (50.59)	<0.0001
Age (years)	57 [49, 63]	57 [50, 63]	56 [49, 62]	<0.0001
Ethnic (%)				<0.0001
White	69,999 (95.72)	105,058 (95.57)	7251 (93.38)	
Others	2945 (4.03)	4596 (4.18)	480 (6.18)	
Townsend deprivation index	−2.46 [−3.81, −0.20]	−2.30 [−3.72, 0.20]	−1.87 [−3.53, 1.19]	<0.0001
Educational level (%)				<0.0001
College or university degree	26,911 (36.80)	34,553 (31.43)	2077 (26.75)	
A levels/AS levels or equivalent and O levels/GCSE or equivalent	27,587 (37.72)	43,212 (39.31)	3166 (40.77)	
Professional qualifications	8022 (10.97)	13,149 (11.96)	956 (12.31)	
No qualification	10,085 (13.79)	18,131 (16.49)	1505 (19.38)	
Annual household income (£) (%)				<0.0001
<18,000	11,170 (15.27)	19,726 (17.94)	1695 (21.83)	
18,000–30,999	15,727 (21.51)	24,488 (22.28)	1726 (22.23)	
31,000–51,999	17,535 (23.98)	25,989 (23.64)	1715 (22.09)	
52,000–100,000	15,145 (20.71)	20,107 (18.29)	1236 (15.92)	
>100,000	4236 (5.79)	5058 (4.60)	274 (3.53)	
Summed MET minutes per week (%)				<0.0001
Low	21,909 (29.96)	39,613 (36.03)	3370 (43.40)	
moderate	31,013 (42.41)	43,738 (39.79)	2828 (36.42)	
High	20,207 (27.63)	26,582 (24.18)	1567 (20.18)	
Body mass index (kg/m^2^)	25.80 [23.45, 28.56]	26.83 [24.31, 29.85]	28.43 [25.57, 31.89]	<0.0001
Alcohol intake (%)				<0.0001
Never	3053 (4.17)	3867 (3.52)	272 (3.50)	
Past	2151 (2.94)	3396 (3.09)	316 (4.07)	
Current	67,883 (92.83)	102,600 (93.33)	7171 (92.35)	
Smoking status (%)				<0.0001
Never	44,518 (60.88)	58,527 (53.24)	3441 (44.31)	
Past	23,014 (31.47)	38,494 (35.02)	2991 (38.52)	
Current	5425 (7.42)	12,596 (11.46)	1315 (16.93)	
Hypertension (%)	29,730 (40.65)	48,961 (44.54)	3668 (47.24)	<0.0001
Family of diabetes (%)	9849 (13.47)	15,411 (14.02)	1185 (15.26)	<0.0001
Family of cardiovascular disease (%)	19,883 (27.19)	29,360 (26.71)	1909 (24.58)	<0.0001
Use of lipid-lowering drugs	6758 (9.24)	12,725 (11.58)	1101 (14.18)	

Data are presented as frequency (%) or median (Q25–Q75). *p* for Kruskal–Wallis tests (continuous variables) or Chi-squared tests (categorical variables). Abbreviations: CSE, Certificate of Secondary Education; GCSE, General Certificate of Secondary Education; HNC, Higher National Certificate; MET, metabolic equivalent; NVQ, National Vocational Qualification.

**Table 2:** Associations of sleep pattern and healthy sleep score with the risks of CMDs and CMM.

Categories	No. of Cases	Incidence (Per 1000 Person-Years)	Crude HR (95% CI)	Adjusted HR * (95% CI)
CMM				
Healthy sleep (score ≥ 4)	783	0.80	1.00 (Reference)	1.00 (Reference)
Intermediate sleep (2 ≤ score ≤ 3)	1739	1.19	1.427 (1.311, 1.552)	1.201 (1.103, 1.308)
Poor sleep (score ≤ 1)	218	2.13	2.545 (2.190, 2.958)	1.653 (1.419, 1.926)
Per 1 decrement of healthy sleep score	2740	1.08	1.306 (1.257, 1.357)	1.151 (1.108, 1.196)
Total CMDs				
Healthy sleep (score ≥ 4)	7770	8.26	1.00 (Reference)	1.00 (Reference)
Intermediate sleep (2 ≤ score ≤ 3)	15,307	11.03	1.289 (1.254, 1.324)	1.148 (1.117, 1.180)
Poor sleep (score ≤ 1)	1525	16.11	1.889 (1.788, 1.995)	1.430 (1.352, 1.512)
Per 1 decrement of healthy sleep score	24,602	10.15	1.198 (1.182, 1.213)	1.105 (1.090, 1.119)
Individual CMDs				
T2DM				
Healthy sleep (score ≥ 4)	2329	2.40	1.00 (Reference)	1.00 (Reference)
Intermediate sleep (2 ≤ score ≤ 3)	5175	3.59	1.449 (1.380, 1.522)	1.155 (1.099, 1.213)
Poor sleep (score ≤ 1)	699	7.04	2.793 (2.566, 3.039)	1.613 (1.479, 1.758)
Per 1 decrement of healthy sleep score	8203	3.26	1.341 (1.311, 1.370)	1.143 (1.118, 1.168)
Stroke				
Healthy sleep (score ≥ 4)	1562	1.60	1.00 (Reference)	1.00 (Reference)
Intermediate sleep (2 ≤ score ≤ 3)	2803	1.92	1.158 (1.088, 1.232)	1.106 (1.039, 1.177)
Poor sleep (score ≤ 1)	229	2.24	1.393 (1.213, 1.601)	1.240 (1.078, 1.427)
Per 1 decrement of healthy sleep score	4594	1.81	1.097 (1.064, 1.130)	1.060 (1.029, 1.093)
CHD				
Healthy sleep (score ≥ 4)	4696	4.91	1.00 (Reference)	1.00 (Reference)
Intermediate sleep (2 ≤ score ≤ 3)	9156	6.45	1.262 (1.218, 1.307)	1.169 (1.128, 1.211)
Poor sleep (score ≤ 1)	831	8.41	1.651 (1.533, 1.777)	1.371 (1.272, 1.477)
Per 1 decrement of healthy sleep score	14,683	5.93	1.162 (1.143, 1.182)	1.101 (1.083, 1.120)

Crude HR (95% CI) adjusted for age, sex, and ethnicity; * HR (95% CI) adjusted for age, sex, ethnicity, Townsend deprivation index, education attainment, annual household income, alcohol intake, physical activity, smoking status, BMI, hypertension, history of diabetes, and cardiovascular disease. Abbreviations: CHD, coronary heart disease; CI, confidence interval; CMD, cardiometabolic disease; CMM, cardiometabolic multimorbidity; HR, hazard ratio; T2DM, type 2 diabetes mellitus.

## Data Availability

The data presented in this study are openly available in the UK Biobank (https://www.ukbiobank.ac.uk/).

## Supplementary Materials

The following supporting information can be downloaded at: https://www.mdpi.com/article/10.3390/healthcare14152296/s1, Figure S1: Flowchart of the selection of study participants from the UK Biobank; Figure S2: Analyzing process of the mediation effects; Table S1: Data fields used in the UK Biobank to identify the outcomes; Table S2: Number and percentage of missing data for covariates; Table S3: Associations between sleep pattern with transitions from baseline to CMDs, CMM and death; Table S4: Linear associations of healthy sleep score with 168 circulating metabolites; Table S5: Association between healthy sleep score-related circulating metabolites and outcomes; Table S6: 14 metabolites associated with all 3 CMDs and CMM; Table S7: Mediating effects of the circulating metabolites both associated with healthy sleep score and outcome; Table S8: Sensitivity analysis of Associations of sleep pattern with the risks of CMDs and CMM; Table S9: Associations of sleep pattern and sleep score with the risks of cardiometabolic disease and cardiometabolic multimorbidity with weighted/unweighted sleep score; Table S10: Associations of sleep pattern and sleep score with the risks of cardiometabolic disease and cardiometabolic multimorbidity, adjusted/not adjusted for BMI.
