# Supplementary material for: Association Between Sleep Patterns and the Risk of Cardiometabolic Diseases or Cardiometabolic Multimorbidity and the Mediating Role of Metabolic Biomarkers: A Prospective Study of the UK Biobank Cohort
Source: Healthcare (Basel). 2026 Jul 29;14(15):2296. doi: 10.3390/healthcare14152296 (PMC13464970; doi:10.3390/healthcare14152296)
Supplement: Supplementary file 1 [file healthcare-14-02296-s001.zip › healthcare-4398283-supplementary.pdf]

Figure S1 Flowchart of the selection of study participants from the UK Biobank

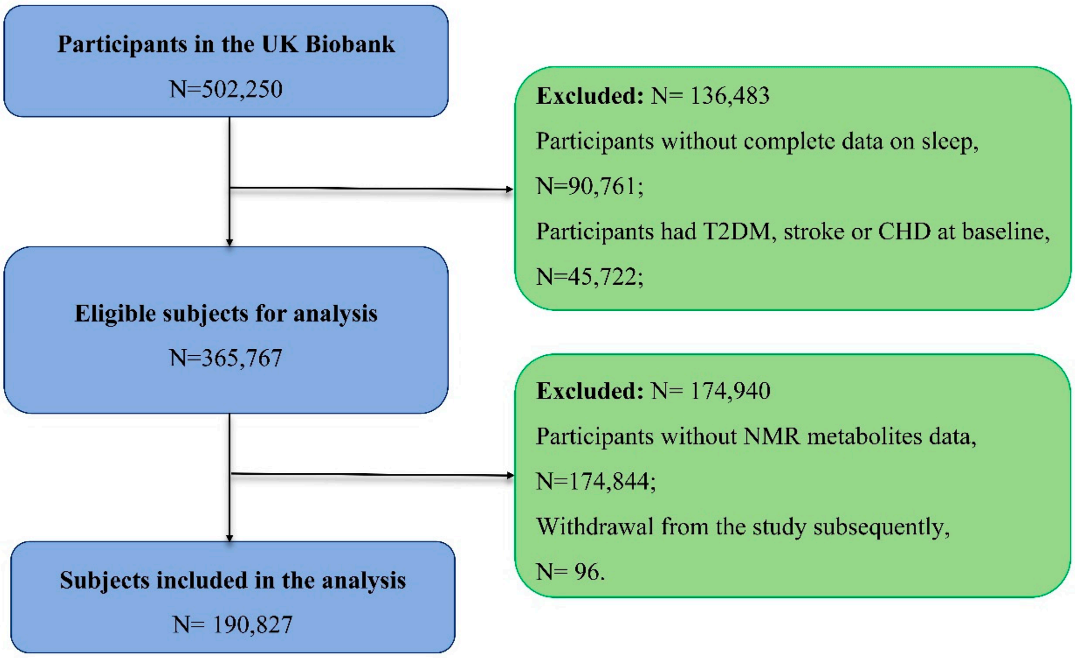

Abbreviations: CHD, coronary heart disease; NMR, nuclear magnetic resonance; T2DM, type 2 diabetes mellitus.

Figure S2 Analyzing process of the mediation effects

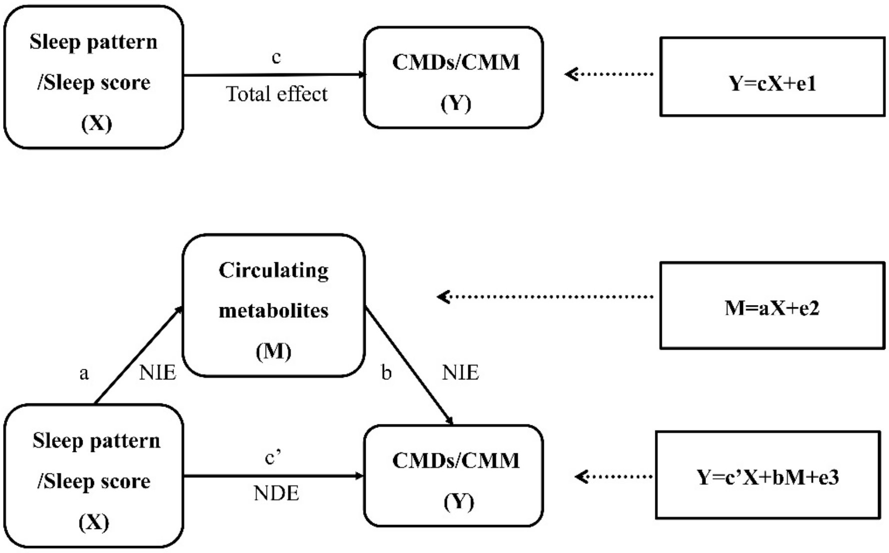

CMD, cardiometabolic disease; CMM, cardiometabolic multimorbidity; NDE, the natural direct effect; NIE, the natural indirect effect.

Table S1 Data fields used in the UK Biobank to identify the outcomes.

Outcomes	Definitions
T2DM	ICD-10: E11
CHD	ICD-10: I20-25; 42000
Stroke	ICD-10: I60-64, I69, G45, G46; 42006, 42008, 131366, 131368
CMM	Refers to the same individual suffering from any two of the following cardiometabolic diseases: T2DM, CHD, and stroke

Abbreviations: ICD-10, the tenth edition of the International Classification of Diseases; T2DM, type 2 diabetes mellitus; CHD, coronary heart disease; CMM, cardiometabolic multimorbidity.

Table S2 Number and percentage of missing data for covariates.

Covariates	No. of missing values	Proportion (%)
Age	0	0
Sex	0	0
Ethnicity	498	0.26

Townsend deprivation index	229	0.12
Education attainment	1,473	0.77
Annual household income	25,000	13.1
Alcohol intake	118	0.06
Physical activity	38,284	20.06
Smoking status	506	0.27
Body mass index	548	0.29
Hypertension	11,727	6.15
History of diabetes	0	0
History of cardiovascular disease	0	0

Table S3 Associations between sleep pattern with transitions from baseline to CMDs, CMM and death.

		**sleep pattern/healthy sleep score**	
	**Events**	**Healthy sleep (score ≥ 4)**	**Per 1 decrement of healthy sleep score**
**Transition pattern A**			
Baseline–> FCMD	24,602	1.00 (Reference)	1.1044 (1.0903 - 1.1187)
Baseline–> Death	8,745	1.00 (Reference)	1.0526 (1.0303 - 1.0754)
FCMD–> CMM	2,740	1.00 (Reference)	1.0362 (0.9933 - 1.08107)
FCMD–> Death	3,840	1.00 (Reference)	0.9679 (0.9323 - 1.0049)
CMM–> Death	717	1.00 (Reference)	0.8970 (0.8244 - 0.9761)
**Transition pattern B**			
Baseline–> T2D	7,366	1.00 (Reference)	1.1730 (1.1470 - 1.2000)
Baseline–> Stroke	3,947	1.00 (Reference)	1.0683 (1.0350 - 1.1030)
Baseline–> CHD	13,289	1.00 (Reference)	1.0852 (1.0660 - 1.1040)
Baseline–> Death	8,745	1.00 (Reference)	1.0362 (1.0140 - 1.0590)
T2D–> CMM	1,221	1.00 (Reference)	0.9902 (0.9230 - 1.0624)
T2D–> Death	1,021	1.00 (Reference)	0.9912 (0.9320 - 1.0540)
Stroke–> CMM	490	1.00 (Reference)	1.0679 (0.9637 - 1.1835)
Stroke–> Death	1,162	1.00 (Reference)	0.9448 (0.8842 - 1.0100)
CHD–> CMM	1,029	1.00 (Reference)	1.0342 (0.9711 - 1.1015)
CHD–> Death	2,374	1.00 (Reference)	0.9626 (0.9152 - 1.0130)

Abbreviations: T2DM, type 2 diabetes mellitus; CHD, coronary heart disease; CMM, cardiometabolic multimorbidity.

Table S4 Linear associations of healthy sleep score with 168 circulating metabolites.

**Metabolites**	**Beta**	**SE**	***P* value**	***FDR***
Total Cholesterol	0.021	0.002	5.82E-19	8.44E-19
Total Cholesterol Minus HDL-C	0.028	0.002	5.36E-31	9.90E-31

Remnant Cholesterol (Non-HDL, Non-LDL -Cholesterol)	0.028	0.002	3.40E-31	6.49E-31
VLDL Cholesterol	0.037	0.002	8.25E-56	2.89E-55
Clinical LDL Cholesterol	0.023	0.002	3.40E-21	5.10E-21
LDL Cholesterol	0.027	0.002	1.38E-28	2.35E-28
HDL Cholesterol	-0.012	0.002	2.21E-08	2.79E-08
Total Triglycerides	0.044	0.002	9.94E-85	1.52E-83
Triglycerides in VLDL	0.043	0.002	5.73E-84	6.87E-83
Triglycerides in LDL	0.044	0.002	2.81E-82	2.62E-81
Triglycerides in HDL	0.032	0.002	8.23E-42	2.03E-41
Total Phospholipids in Lipoprotein Particles	0.026	0.002	7.63E-30	1.35E-29
Phospholipids in VLDL	0.043	0.002	4.54E-78	2.82E-77
Phospholipids in LDL	0.028	0.002	4.73E-31	8.83E-31
Phospholipids in HDL	-0.002	0.002	0.376509	0.392879
Total Esterified Cholesterol	0.019	0.002	1.76E-16	2.50E-16
Cholesteryl Esters in VLDL	0.033	0.002	4.06E-43	1.07E-42
Cholesteryl Esters in LDL	0.030	0.002	7.92E-36	1.66E-35
Cholesteryl Esters in HDL	-0.013	0.002	1.38E-09	1.80E-09
Total Free Cholesterol	0.024	0.002	4.92E-25	7.95E-25
Free Cholesterol in VLDL	0.041	0.002	8.17E-72	3.81E-71
Free Cholesterol in LDL	0.017	0.002	5.71E-12	7.93E-12
Free Cholesterol in HDL	-0.008	0.002	0.000186	0.000214
Total Lipids in Lipoprotein Particles	0.035	0.002	2.51E-49	7.67E-49
Total Lipids in VLDL	0.044	0.002	6.86E-83	7.20E-82
Total Lipids in LDL	0.029	0.002	8.20E-34	1.62E-33
Total Lipids in HDL	-0.004	0.002	0.034164	0.03776
Total Concentration of Lipoprotein Particles	0.015	0.002	6.80E-11	9.14E-11
Concentration of VLDL Particles	0.040	0.002	1.24E-64	4.83E-64
Concentration of LDL Particles	0.030	0.002	5.63E-36	1.20E-35
Concentration of HDL Particles	0.010	0.002	2.69E-06	3.32E-06
Average Diameter for VLDL Particles	0.039	0.002	1.30E-75	6.83E-75
Average Diameter for LDL Particles	-0.012	0.002	7.48E-08	9.38E-08
Average Diameter for HDL Particles	-0.030	0.002	1.87E-51	5.91E-51
Phosphoglycerides	0.024	0.002	6.04E-26	9.85E-26
Total Cholines	0.021	0.002	1.59E-20	2.37E-20
Phosphatidylcholines	0.019	0.002	5.72E-17	8.22E-17
Sphingomyelins	0.015	0.002	7.77E-12	1.07E-11
Apolipoprotein B	0.030	0.002	9.29E-36	1.93E-35
Apolipoprotein A1	0.002	0.002	0.440988	0.457321
Total Fatty Acids	0.044	0.002	2.87E-79	2.01E-78
Degree of Unsaturation	-0.025	0.002	1.00E-29	1.76E-29
Omega-3 Fatty Acids	0.016	0.002	1.29E-11	1.75E-11
Omega-6 Fatty Acids	0.029	0.002	3.91E-35	7.81E-35

Polyunsaturated Fatty Acids	0.029	0.002	2.21E-35	4.48E-35
Monounsaturated Fatty Acids	0.049	0.002	4.24E-102	7.12E-100
Saturated Fatty Acids	0.044	0.002	1.56E-80	1.19E-79
Linoleic Acid	0.023	0.002	2.88E-21	4.36E-21
Docosahexaenoic Acid	0.000	0.002	0.898838	0.898838
Alanine	0.011	0.002	9.15E-06	1.11E-05
Glutamine	-0.011	0.002	9.34E-06	1.12E-05
Glycine	-0.004	0.002	0.04707	0.051685
Histidine	-0.002	0.002	0.45066	0.464484
Total Concentration of Branched-Chain Amino Acids (Leucine + Isoleucine + Valine)	0.009	0.002	4.89E-05	5.71E-05
Isoleucine	0.006	0.002	0.017123	0.019051
Leucine	0.008	0.002	0.000215	0.000245
Valine	0.011	0.002	2.86E-06	3.51E-06
Phenylalanine	-0.004	0.002	0.062327	0.067121
Tyrosine	0.017	0.002	9.79E-13	1.37E-12
Glucose	0.010	0.002	3.20E-05	3.76E-05
Lactate	0.007	0.002	0.004477	0.005014
Pyruvate	0.002	0.002	0.318853	0.334795
Citrate	-0.031	0.002	9.90E-40	2.31E-39
3-Hydroxybutyrate	-0.001	0.002	0.585383	0.59966
Acetate	-0.011	0.002	1.12E-05	1.32E-05
Acetoacetate	0.005	0.002	0.047982	0.052344
Acetone	-0.004	0.002	0.131365	0.138801
Creatinine	0.012	0.002	5.90E-09	7.50E-09
Albumin	0.000	0.002	0.879496	0.884762
Glycoprotein Acetyls	0.047	0.002	1.25E-98	1.05E-96
Concentration of Chylomicrons and Extremely Large VLDL Particles	0.041	0.002	1.59E-76	8.88E-76
Total Lipids in Chylomicrons and Extremely Large VLDL	0.041	0.002	1.59E-73	7.64E-73
Phospholipids in Chylomicrons and Extremely Large VLDL	0.041	0.002	1.05E-76	6.06E-76
Cholesterol in Chylomicrons and Extremely Large VLDL	0.043	0.002	4.82E-81	3.86E-80
Cholesteryl Esters in Chylomicrons and Extremely Large VLDL	0.043	0.002	1.56E-81	1.38E-80
Free Cholesterol in Chylomicrons and Extremely Large VLDL	0.042	0.002	5.50E-78	3.30E-77
Triglycerides in Chylomicrons and Extremely Large VLDL	0.039	0.002	3.06E-68	1.32E-67
Concentration of Very Large VLDL	0.044	0.002	6.87E-89	3.85E-87

Particles				
Total Lipids in Very Large VLDL	0.044	0.002	2.88E-87	6.92E-86
Phospholipids in Very Large VLDL	0.044	0.002	1.55E-86	3.25E-85
Cholesterol in Very Large VLDL	0.043	0.002	2.05E-82	2.03E-81
Cholesteryl Esters in Very Large VLDL	0.041	0.002	5.42E-75	2.76E-74
Free Cholesterol in Very Large VLDL	0.044	0.002	5.45E-85	9.15E-84
Triglycerides in Very Large VLDL	0.043	0.002	1.95E-84	2.65E-83
Concentration of Large VLDL Particles	0.044	0.002	1.10E-85	2.05E-84
Total Lipids in Large VLDL	0.043	0.002	1.67E-83	1.87E-82
Phospholipids in Large VLDL	0.044	0.002	1.39E-87	3.90E-86
Cholesterol in Large VLDL	0.042	0.002	8.19E-79	5.50E-78
Cholesteryl Esters in Large VLDL	0.039	0.002	3.31E-66	1.36E-65
Free Cholesterol in Large VLDL	0.044	0.002	3.07E-88	1.29E-86
Triglycerides in Large VLDL	0.042	0.002	1.10E-78	7.13E-78
Concentration of Medium VLDL Particles	0.038	0.002	2.75E-58	1.03E-57
Total Lipids in Medium VLDL	0.040	0.002	6.34E-67	2.66E-66
Phospholipids in Medium VLDL	0.037	0.002	6.01E-55	2.06E-54
Cholesterol in Medium VLDL	0.023	0.002	2.65E-22	4.05E-22
Cholesteryl Esters in Medium VLDL	0.014	0.002	2.17E-09	2.80E-09
Free Cholesterol in Medium VLDL	0.033	0.002	1.25E-43	3.34E-43
Triglycerides in Medium VLDL	0.043	0.002	2.69E-81	2.26E-80
Concentration of Small VLDL Particles	0.041	0.002	1.82E-70	8.05E-70
Total Lipids in Small VLDL	0.041	0.002	9.16E-71	4.16E-70
Phospholipids in Small VLDL	0.037	0.002	4.20E-57	1.50E-56
Cholesterol in Small VLDL	0.034	0.002	1.00E-47	3.01E-47
Cholesteryl Esters in Small VLDL	0.035	0.002	2.56E-50	7.98E-50
Free Cholesterol in Small VLDL	0.032	0.002	1.18E-41	2.86E-41
Triglycerides in Small VLDL	0.042	0.002	1.88E-74	9.30E-74
Concentration of Very Small VLDL Particles	0.026	0.002	1.62E-28	2.72E-28
Total Lipids in Very Small VLDL	0.026	0.002	2.55E-29	4.42E-29
Phospholipids in Very Small VLDL	0.028	0.002	2.56E-32	5.01E-32
Cholesterol in Very Small VLDL	0.015	0.002	7.15E-11	9.53E-11
Cholesteryl Esters in Very Small VLDL	0.011	0.002	1.67E-06	2.07E-06
Free Cholesterol in Very Small VLDL	0.024	0.002	1.41E-24	2.26E-24
Triglycerides in Very Small VLDL	0.040	0.002	1.55E-65	6.20E-65
Concentration of IDL Particles	0.018	0.002	1.06E-13	1.49E-13
Total Lipids in IDL	0.015	0.002	3.70E-10	4.89E-10
Phospholipids in IDL	0.015	0.002	4.91E-10	6.44E-10
Cholesterol in IDL	0.010	0.002	1.05E-05	1.25E-05
Cholesteryl Esters in IDL	0.011	0.002	5.71E-06	6.95E-06
Free Cholesterol in IDL	0.009	0.002	8.85E-05	0.000103
Triglycerides in IDL	0.039	0.002	9.31E-63	3.56E-62
Concentration of Large LDL Particles	0.027	0.002	4.36E-29	7.47E-29

Total Lipids in Large LDL	0.024	0.002	1.74E-24	2.75E-24
Phospholipids in Large LDL	0.023	0.002	1.25E-22	1.92E-22
Cholesterol in Large LDL	0.022	0.002	8.81E-20	1.29E-19
Cholesteryl Esters in Large LDL	0.024	0.002	2.06E-24	3.23E-24
Free Cholesterol in Large LDL	0.014	0.002	5.36E-09	6.87E-09
Triglycerides in Large LDL	0.043	0.002	3.03E-76	1.64E-75
Concentration of Medium LDL Particles	0.033	0.002	7.57E-44	2.05E-43
Total Lipids in Medium LDL	0.036	0.002	1.61E-51	5.21E-51
Phospholipids in Medium LDL	0.034	0.002	1.05E-46	2.99E-46
Cholesterol in Medium LDL	0.034	0.002	5.91E-47	1.71E-46
Cholesteryl Esters in Medium LDL	0.038	0.002	1.96E-57	7.15E-57
Free Cholesterol in Medium LDL	0.022	0.002	8.20E-20	1.21E-19
Triglycerides in Medium LDL	0.046	0.002	7.03E-88	2.36E-86
Concentration of Small LDL Particles	0.032	0.002	7.09E-42	1.78E-41
Total Lipids in Small LDL	0.034	0.002	1.87E-45	5.14E-45
Phospholipids in Small LDL	0.028	0.002	1.23E-31	2.37E-31
Cholesterol in Small LDL	0.032	0.002	1.59E-41	3.82E-41
Cholesteryl Esters in Small LDL	0.037	0.002	3.00E-54	1.01E-53
Free Cholesterol in Small LDL	0.016	0.002	1.03E-11	1.40E-11
Triglycerides in Small LDL	0.044	0.002	2.05E-84	2.65E-83
Concentration of Very Large HDL Particles	-0.022	0.002	1.09E-26	1.81E-26
Total Lipids in Very Large HDL	-0.027	0.002	5.25E-38	1.16E-37
Phospholipids in Very Large HDL	-0.028	0.002	4.84E-42	1.23E-41
Cholesterol in Very Large HDL	-0.027	0.002	1.46E-38	3.36E-38
Cholesteryl Esters in Very Large HDL	-0.027	0.002	2.04E-38	4.62E-38
Free Cholesterol in Very Large HDL	-0.027	0.002	1.92E-35	3.94E-35
Triglycerides in Very Large HDL	0.024	0.002	2.62E-23	4.07E-23
Concentration of Large HDL Particles	-0.023	0.002	4.86E-30	8.68E-30
Total Lipids in Large HDL	-0.023	0.002	4.59E-30	8.29E-30
Phospholipids in Large HDL	-0.021	0.002	4.15E-26	6.84E-26
Cholesterol in Large HDL	-0.025	0.002	1.10E-36	2.39E-36
Cholesteryl Esters in Large HDL	-0.026	0.002	3.87E-38	8.67E-38
Free Cholesterol in Large HDL	-0.023	0.002	4.24E-31	8.01E-31
Triglycerides in Large HDL	0.008	0.002	0.000722	0.000814
Concentration of Medium HDL Particles	0.001	0.002	0.650622	0.662451
Total Lipids in Medium HDL	0.004	0.002	0.04969	0.053857
Phospholipids in Medium HDL	0.008	0.002	0.000495	0.000562
Cholesterol in Medium HDL	-0.003	0.002	0.125549	0.133495
Cholesteryl Esters in Medium HDL	-0.004	0.002	0.063201	0.067629
Free Cholesterol in Medium HDL	0.000	0.002	0.862019	0.872405
Triglycerides in Medium HDL	0.032	0.002	2.06E-41	4.87E-41
Concentration of Small HDL Particles	0.034	0.002	8.61E-46	2.41E-45
Total Lipids in Small HDL	0.036	0.002	1.98E-53	6.53E-53
Phospholipids in Small HDL	0.034	0.002	3.50E-47	1.03E-46

Cholesterol in Small HDL	0.030	0.002	4.51E-36	9.72E-36
Cholesteryl Esters in Small HDL	0.028	0.002	6.39E-31	1.17E-30
Free Cholesterol in Small HDL	0.032	0.002	2.10E-42	5.42E-42
Triglycerides in Small HDL	0.042	0.002	1.88E-80	1.37E-79

Abbreviations: HDL, high-density lipoprotein; IDL, intermediate-density lipoprotein; LDL, low-density lipoprotein; VLDL, very low-density lipoprotein.

Table S5 Association between healthy sleep score-related circulating metabolites and outcomes.

Sleep-related metabolites	T2DM					CHD					Stroke					CMM				
	HR	95% Low	95% Up	P value	FDR	HR	95% Low	95% Up	P value	FDR	HR	95% Low	95% Up	P value	FDR	HR	95% Low	95% Up	P value	FDR
Total Cholesterol	0.910	0.889	0.930	1.78E-16	2.55E-16	1.045	1.028	1.062	1.77E-07	2.15E-07	0.980	0.951	1.009	1.80E-01	3.62E-01	0.961	0.925	0.999	4.32E-02	5.42E-02
Total Cholesterol Minus HDL-C	0.991	0.970	1.013	4.35E-01	4.41E-01	1.090	1.073	1.108	3.29E-26	6.49E-26	0.987	0.959	1.016	3.90E-01	5.41E-01	1.024	0.987	1.063	2.07E-01	2.40E-01
Remnant Cholesterol (Non-HD	1.017	0.995	1.039	1.38E-01	1.43E-01	1.101	1.083	1.119	4.42E-32	1.03E-31	1.000	0.971	1.029	9.85E-01	9.91E-01	1.045	1.007	1.085	1.98E-02	2.53E-02

L, Non-LDL-Cholesterol)																				
VLDL Cholesterol	1.171	1.147	1.197	1.01E-48	1.97E-48	1.133	1.115	1.152	9.47E-53	6.26E-52	0.997	0.968	1.027	8.29E-01	9.01E-01	1.136	1.095	1.179	1.17E-11	2.54E-11
Clinical LDL Cholesterol	0.916	0.896	0.936	3.74E-15	5.31E-15	1.070	1.053	1.087	7.70E-17	1.11E-16	0.982	0.954	1.010	2.07E-01	3.87E-01	0.973	0.937	1.010	1.47E-01	1.75E-01
LDL Cholesterol	0.969	0.948	0.990	4.04E-03	4.42E-03	1.077	1.059	1.094	1.31E-19	2.03E-19	0.976	0.948	1.005	1.06E-01	2.87E-01	1.004	0.967	1.042	8.32E-01	8.66E-01
HDL Cholesterol	0.668	0.647	0.689	9.04E-145	2.69E-144	0.854	0.837	0.872	2.98E-50	1.62E-49	0.970	0.937	1.004	8.14E-02	2.38E-01	0.751	0.713	0.791	1.65E-27	6.27E-27
Total Triglyce	1.40	1.382	1.430	0.00E+00	0.00E+00	1.11	1.093	1.127	2.14	6.26	0.98	0.954	1.014	2.79E	4.19E	1.24	1.209	1.289	1.96	2.98

rides	6					0			E-39	E-39	3			-01	-01	9			E-41	E-40
Triglycerides in VLDL	1.405	1.381	1.430	0.00E+00	0.00E+00	1.104	1.087	1.122	3.44E-35	8.57E-35	0.973	0.943	1.003	7.75E-02	2.35E-01	1.240	1.200	1.282	4.53E-38	4.59E-37
Triglycerides in LDL	1.362	1.340	1.384	0.00E+00	0.00E+00	1.134	1.118	1.151	1.12E-61	2.43E-60	1.030	1.000	1.060	4.81E-02	1.74E-01	1.262	1.224	1.301	3.13E-51	1.19E-49
Triglycerides in HDL	1.349	1.326	1.373	2.82E-243	1.48E-242	1.085	1.068	1.102	2.80E-24	5.07E-24	1.018	0.988	1.048	2.43E-01	3.95E-01	1.223	1.184	1.264	1.98E-33	1.31E-32
Total Phospholipids in Lipoprotein Particles	1.042	1.018	1.066	3.98E-04	4.59E-04	1.032	1.015	1.050	2.78E-04	3.21E-04	0.980	0.951	1.011	2.00E-01	3.80E-01	1.026	0.986	1.067	2.08E-01	2.40E-01
Phospholipids in VL	1.319	1.293	1.345	4.67E-163	1.51E-162	1.138	1.120	1.156	2.44E	2.32E	0.991	0.961	1.021	5.47E-	6.82E-	1.213	1.171	1.257	2.26E	7.99E

DL									-56	-55				01	01				-26	-26
Phospholipids in LDL	0.982	0.961	1.004	1.02E-01	1.07E-01	1.084	1.067	1.102	2.18E-23	3.64E-23	0.982	0.954	1.011	2.24E-01	3.95E-01	1.016	0.979	1.055	4.00E-01	4.37E-01
Total Esterified Cholesterol	0.890	0.870	0.910	5.66E-24	8.87E-24	1.035	1.018	1.053	3.88E-05	4.54E-05	0.978	0.949	1.008	1.43E-01	3.28E-01	0.945	0.909	0.983	4.55E-03	6.06E-03
Cholesteryl Esters in VLDL	1.096	1.073	1.120	7.10E-17	1.04E-16	1.128	1.110	1.146	1.72E-49	9.02E-49	1.003	0.974	1.033	8.43E-01	9.01E-01	1.097	1.057	1.139	9.70E-07	1.68E-06
Cholesteryl Esters in LDL	1.007	0.986	1.029	5.09E-01	5.12E-01	1.084	1.067	1.102	2.85E-23	4.71E-23	0.975	0.947	1.004	8.67E-02	2.44E-01	1.027	0.990	1.066	1.55E-01	1.83E-01
Cholesteryl Esters in HDL	0.646	0.627	0.666	5.38E-170	1.82E-169	0.846	0.829	0.864	4.05E-57	4.40E-56	0.964	0.931	0.998	3.57E-02	1.36E-01	0.731	0.695	0.770	1.10E-32	1.19E-32
Tota	0	0.	0.	4.70	5.37	1	1.	1.	4	5	0	0.	1.	3.	4.	1	0.	1.	9	9

l Free Cholesterol	.961	940	983	E-04	E-04	.067	050	085	.26E-15	.89E-15	.985	957	014	17E-01	55E-01	.000	963	039	.81E-01	.84E-01
Free Cholesterol in VLDL	1.266	1.240	1.292	2.68 E-112	6.68 E-112	1.135	1.117	1.153	9.95E-54	7.56E-53	0.988	0.959	1.018	4.45E-01	6.03E-01	1.182	1.140	1.226	1.66E-19	4.85E-19
Free Cholesterol in LDL	0.871	0.852	0.890	8.11 E-35	1.42 E-34	1.053	1.036	1.070	2.23E-10	2.87E-10	0.981	0.953	1.010	1.94E-01	3.74E-01	0.943	0.909	0.979	2.28E-03	3.12E-03
Free Cholesterol in HDL	0.777	0.754	0.801	7.49 E-61	1.60 E-60	0.895	0.877	0.914	4.30E-26	8.38E-26	0.993	0.959	1.028	6.89E-01	8.00E-01	0.844	0.803	0.888	5.46E-11	1.15E-10
Total Lipids in Lipoprotein Particles	1.120	1.096	1.143	1.00 E-25	1.60 E-25	1.073	1.056	1.090	1.24E-17	1.85E-17	0.978	0.950	1.007	1.40E-01	3.28E-01	1.081	1.042	1.121	3.55E-05	5.80E-05
Total Lipids in	1.36	1.335	1.387	3.15 E-218	1.29 E-217	1.12	1.109	1.145	1.29	6.54	0.98	0.953	1.012	2.40E	3.95E	1.22	1.183	1.269	2.10	9.39

VLDL	1					6			E-48	E-48	2			-01	-01	5			E-30	E-30
Total Lipids in LDL	1.002	0.981	1.024	8.57E-01	8.57E-01	1.086	1.069	1.103	4.89E-24	8.74E-24	0.980	0.952	1.009	1.84E-01	3.62E-01	1.028	0.990	1.066	1.52E-01	1.80E-01
Total Lipids in HDL	0.811	0.789	0.835	1.54E-47	2.89E-47	0.882	0.864	0.899	5.15E-35	1.26E-34	0.976	0.943	1.009	1.56E-01	3.43E-01	0.841	0.801	0.883	2.19E-12	5.04E-12
Total Concentration of Lipoprotein Particles	0.883	0.861	0.905	1.28E-23	1.99E-23	0.933	0.917	0.950	3.90E-14	5.25E-14	0.944	0.915	0.975	3.98E-04	5.50E-03	0.879	0.843	0.917	2.21E-09	4.25E-09
Concentration of VLDL Particles	1.260	1.234	1.286	8.74E-109	2.14E-108	1.142	1.124	1.160	1.03E-59	1.74E-58	1.001	0.972	1.031	9.54E-01	9.67E-01	1.193	1.151	1.237	8.66E-22	2.69E-21
Concentration of LDL	1.027	1.005	1.049	1.46E-02	1.57E-02	1.111	1.093	1.128	6.21E	1.75E	0.990	0.962	1.019	4.99E-	6.57E-	1.057	1.018	1.096	3.33E	4.48E

Particles									-39	-38				01	01				-03	-03
Concentration of HDL Particles	0.870	0.849	0.892	3.31E-28	5.41E-28	0.910	0.894	0.927	2.53E-24	4.63E-24	0.942	0.913	0.973	2.84E-04	4.31E-03	0.861	0.825	0.899	9.20E-12	2.03E-11
Average Diameter for VLDL Particles	1.526	1.489	1.563	2.92E-251	1.59E-250	1.077	1.058	1.097	8.32E-16	1.17E-15	0.925	0.895	0.956	2.81E-06	1.07E-04	1.228	1.178	1.281	7.22E-22	2.29E-21
Average Diameter for LDL Particles	0.774	0.758	0.789	1.57E-137	4.34E-137	1.012	0.995	1.028	1.67E-01	1.76E-01	1.017	0.987	1.048	2.63E-01	4.08E-01	0.893	0.862	0.926	8.87E-10	1.75E-09
Average Diameter for HDL Particles	0.660	0.639	0.682	1.89E-139	5.42E-139	0.866	0.848	0.885	7.34E-40	2.19E-39	1.039	1.003	1.076	3.52E-02	1.36E-01	0.797	0.756	0.841	5.81E-17	1.50E-16
Phosphoglycerides	1.077	1.053	1.102	1.50E-10	1.90E-10	1.009	0.992	1.027	2.87E-0	3.01E-0	0.978	0.948	1.009	1.54E-01	3.43E-01	1.031	0.990	1.073	1.36E-0	1.64E-0

									1	1									1	1
Total Cholines	1.012	0.988	1.035	3.34E-01	3.41E-01	1.005	0.988	1.023	5.48E-01	5.67E-01	0.975	0.945	1.006	1.11E-01	2.96E-01	0.995	0.956	1.037	8.23E-01	8.63E-01
Phosphatidylcholines	1.036	1.012	1.060	3.40E-03	3.75E-03	1.000	0.982	1.017	9.59E-01	9.59E-01	0.978	0.948	1.009	1.69E-01	3.62E-01	1.006	0.966	1.048	7.68E-01	8.11E-01
Sphingomyelins	0.868	0.847	0.889	1.95E-31	3.26E-31	1.023	1.006	1.041	9.50E-03	1.05E-02	1.003	0.973	1.035	8.32E-01	9.01E-01	0.945	0.908	0.985	6.71E-03	8.87E-03
Apolipoprotein B	1.035	1.013	1.057	1.85E-03	2.07E-03	1.113	1.096	1.131	1.92E-40	5.84E-40	0.995	0.967	1.024	7.48E-01	8.49E-01	1.063	1.025	1.103	1.16E-03	1.63E-03
Total Fatty Acids	1.277	1.253	1.301	3.17E-139	8.92E-139	1.087	1.070	1.105	1.84E-25	3.54E-25	0.982	0.953	1.012	2.29E-01	3.95E-01	1.168	1.129	1.209	7.46E-19	2.10E-18
Degree of	0.6	0.65	0.68	1.58E-257	1.00E-256	0.9	0.89	0.93	6.2	1.1	0.9	0.91	0.97	1.94	3.60	0.7	0.72	0.78	1.8	3.1

Unsaturation	65	0	1			15	9	1	2E-24	0E-23	43	4	2	E-04	E-03	53	4	3	9E-44	9E-43
Omega-3 Fatty Acids	1.063	1.040	1.087	3.25E-08	3.95E-08	1.002	0.985	1.018	8.59E-01	8.65E-01	0.967	0.938	0.996	2.73E-02	1.23E-01	0.992	0.954	1.032	6.92E-01	7.41E-01
Omega-6 Fatty Acids	1.037	1.015	1.060	1.00E-03	1.14E-03	1.059	1.042	1.076	7.27E-12	9.53E-12	0.952	0.924	0.981	1.37E-03	1.30E-02	1.026	0.988	1.065	1.86E-01	2.17E-01
Polyunsaturated Fatty Acids	1.050	1.027	1.073	1.52E-05	1.81E-05	1.051	1.034	1.068	3.92E-09	4.97E-09	0.949	0.921	0.979	7.82E-04	9.91E-03	1.020	0.982	1.060	3.08E-01	3.41E-01
Monounsaturated Fatty Acids	1.344	1.321	1.368	5.88E-241	2.88E-240	1.107	1.090	1.125	2.02E-38	5.48E-38	0.998	0.969	1.028	9.02E-01	9.24E-01	1.221	1.182	1.261	7.15E-34	5.18E-33
Saturated Fatty Acids	1.321	1.298	1.345	7.92E-210	3.17E-209	1.084	1.067	1.101	2.31E-2	4.28E-2	0.998	0.969	1.028	8.87E-01	9.23E-01	1.196	1.158	1.236	3.34E-2	1.21E-2

Linoleic Acid	1.016	0.994	1.038	1.45E-01	1.50E-01	1.056	1.039	1.073	3.85E-11	5.00E-11	0.951	0.923	0.980	8.63E-04	1.01E-02	1.016	0.978	1.054	4.19E-01	4.55E-01
Alanine	1.213	1.188	1.239	6.91E-72	1.50E-71	1.014	0.998	1.031	8.65E-02	9.32E-02	1.002	0.973	1.032	8.93E-01	9.23E-01	1.127	1.086	1.170	2.23E-10	4.58E-10
Glutamine	0.851	0.832	0.870	9.00E-46	1.65E-45	0.996	0.980	1.013	6.36E-01	6.49E-01	1.007	0.978	1.038	6.30E-01	7.60E-01	0.936	0.901	0.973	7.06E-04	1.00E-03
Total Concentration of Branched-Chain Amino Acids (Leucine + Isole	1.236	1.212	1.260	7.90E-101	1.88E-100	1.032	1.015	1.049	2.41E-04	2.80E-04	0.982	0.952	1.013	2.57E-01	4.07E-01	1.110	1.070	1.151	2.04E-08	3.69E-08

ucine + Valine)																				
Isoleucine	1.174	1.151	1.196	1.28E-59	2.67E-59	1.037	1.020	1.054	1.17E-05	1.40E-05	0.998	0.969	1.028	8.91E-01	9.23E-01	1.092	1.054	1.131	1.12E-06	1.91E-06
Leucine	1.181	1.158	1.205	4.22E-60	8.91E-60	1.028	1.011	1.045	1.41E-03	1.57E-03	0.982	0.953	1.013	2.60E-01	4.07E-01	1.074	1.035	1.115	1.43E-04	2.22E-04
Valine	1.283	1.259	1.309	3.18E-140	9.30E-140	1.029	1.012	1.047	7.51E-04	8.58E-04	0.977	0.947	1.008	1.41E-01	3.28E-01	1.133	1.092	1.176	2.61E-11	5.59E-11
Tyrosine	1.213	1.189	1.237	7.78E-80	1.74E-79	1.028	1.011	1.045	9.64E-04	1.09E-03	1.030	1.000	1.060	4.79E-02	1.74E-01	1.120	1.080	1.161	7.25E-10	1.45E-09
Glucose	1.283	1.274	1.292	0.00E+00	0.00E+00	1.003	0.988	1.019	7.01E-01	7.10E-01	1.010	0.982	1.038	4.97E-01	6.57E-01	1.179	1.161	1.196	5.40E-10	8.21E-10

																			2	0
Lactate	1.148	1.125	1.172	1.65E-39	2.99E-39	1.008	0.992	1.025	3.41E-01	3.55E-01	0.995	0.966	1.025	7.55E-01	8.51E-01	1.097	1.057	1.138	7.65E-07	1.34E-06
Citrate	1.029	1.006	1.052	1.32E-02	1.43E-02	0.981	0.964	0.998	2.49E-02	2.70E-02	0.997	0.968	1.027	8.37E-01	9.01E-01	1.025	0.986	1.066	2.17E-01	2.47E-01
Acetate	0.872	0.840	0.905	9.81E-13	1.32E-12	0.987	0.971	1.004	1.36E-01	1.45E-01	0.990	0.961	1.020	5.01E-01	6.57E-01	0.908	0.855	0.964	1.49E-03	2.08E-03
Creatinine	1.064	1.046	1.082	7.35E-13	9.98E-13	1.069	1.055	1.083	1.07E-24	2.01E-24	1.051	1.024	1.079	2.13E-04	3.60E-03	1.084	1.059	1.110	8.80E-12	1.97E-11
Glycoprotein Acetyls	1.384	1.355	1.413	1.10E-201	4.29E-201	1.153	1.134	1.172	9.14E-63	2.32E-61	1.036	1.005	1.069	2.45E-02	1.16E-01	1.279	1.232	1.327	9.23E-39	1.08E-37
Concentratio	1.3	1.35	1.40	0.00E+00	0.00E+00	1.0	1.06	1.10	7.0	1.2	0.9	0.95	1.01	2.49	3.99	1.2	1.18	1.25	2.6	2.1

n of Chylomicrons and Extremely Large VLDL Particles	79	7	1			83	6	0	0E-24	2E-23	82	3	3	E-01	E-01	18	1	7	7E-35	4E-34
Total Lipids in Chylomicrons and Extremely Large VLDL	1.365	1.344	1.387	0.00E+00	0.00E+00	1.077	1.061	1.094	4.44E-21	7.18E-21	0.981	0.952	1.011	2.09E-01	3.87E-01	1.208	1.171	1.246	3.55E-33	2.16E-32
Phospholipids in Chylomicrons and Extremely Larg	1.377	1.355	1.400	9.09080788347894e-321	1.06293283046286e-319	1.082	1.065	1.099	3.79E-23	6.19E-23	0.982	0.953	1.012	2.41E-01	3.95E-01	1.215	1.177	1.254	2.65E-33	1.68E-32

e VLDL																				
Cholesterol in Chylomicrons and Extremely Large VLDL	1.371	1.347	1.396	9.13E-268	6.31E-267	1.095	1.077	1.112	5.61E-29	1.15E-28	0.983	0.953	1.014	2.74E-01	4.16E-01	1.211	1.171	1.252	2.23E-29	9.68E-29
Cholesteryl Esters in Chylomicrons and Extremely Large VLDL	1.362	1.338	1.387	2.82E-240	1.34E-239	1.103	1.085	1.121	2.79E-33	6.73E-33	0.986	0.957	1.017	3.83E-01	5.41E-01	1.209	1.168	1.251	1.10E-27	4.29E-27
Free Cholesterol in Chylomicrons and	1.374	1.351	1.397	6.93E-295	6.58E-294	1.083	1.067	1.101	1.95E-23	3.29E-23	0.979	0.950	1.010	1.78E-01	3.62E-01	1.210	1.171	1.250	1.28E-30	5.90E-30

Extremely Large VLDL Triglycerides in Chylomicrons and Extremely Large VLDL	1.352	1.332	1.372	0.00E+00	0.00E+00	1.069	1.053	1.086	7.45E-18	1.12E-17	0.980	0.951	1.010	1.91E-01	3.71E-01	1.201	1.166	1.237	1.72E-33	1.19E-32
Concentration of Very Large VLDL Particles	1.423	1.398	1.449	1.48219693752374e-321	1.87744945419674e-320	1.118	1.100	1.136	2.86E-42	9.25E-42	0.977	0.947	1.008	1.38E-01	3.28E-01	1.252	1.210	1.295	9.95E-39	1.08E-37
Total Lipids in Very Large VLDL	1.417	1.392	1.443	1.18575755001899e-317	1.28739391850727e-316	1.113	1.095	1.130	3.80E-39	1.09E-38	0.975	0.946	1.006	1.14E-01	3.00E-01	1.246	1.205	1.289	1.14E-37	1.08E-36
Phos	1	1.	1.	2.68	2.26	1	1.	1.	1	6	0	0.	1.	1.	3.	1	1.	1.	1	9

pholipids in Very Large VLDL	.408	382	434	E-289	E-288	.120	102	138	.81E-43	.40E-43	.979	949	010	81E-01	62E-01	.243	202	287	.09E-35	.20E-35
Cholesterol in Very Large VLDL	1.351	1.323	1.378	2.91E-184	1.08E-183	1.137	1.118	1.155	5.06E-53	3.50E-52	0.981	0.951	1.011	2.13E-01	3.91E-01	1.219	1.175	1.264	2.98E-26	1.03E-25
Cholesteryl Esters in Very Large VLDL	1.281	1.254	1.308	6.49E-115	1.64E-114	1.144	1.125	1.163	2.73E-57	3.30E-56	0.983	0.953	1.013	2.70E-01	4.15E-01	1.187	1.143	1.233	4.85E-19	1.39E-18
Free Cholesterol in Very Large VLDL	1.393	1.367	1.420	4.26E-256	2.49E-255	1.124	1.106	1.142	1.65E-45	6.60E-45	0.979	0.949	1.010	1.78E-01	3.62E-01	1.236	1.194	1.280	1.08E-32	6.19E-32
Triglycerides in Very Large VLDL	1.422	1.398	1.446	0.00E+00	0.00E+00	1.099	1.082	1.116	1.01E-31	2.26E-31	0.973	0.944	1.004	8.40E-02	2.41E-01	1.246	1.206	1.287	1.31E-40	1.66E-39

Concentration of Large VLDL Particles	1.402	1.375	1.428	5.47E-266	3.61E-265	1.124	1.106	1.142	1.02E-45	4.31E-45	0.972	0.943	1.003	7.67E-02	2.35E-01	1.242	1.199	1.286	4.06E-34	3.09E-33
Total Lipids in Large VLDL	1.391	1.365	1.418	1.09E-251	6.14E-251	1.120	1.102	1.138	6.25E-43	2.11E-42	0.967	0.938	0.997	3.41E-02	1.36E-01	1.234	1.192	1.278	6.42E-32	3.05E-31
Phospholipids in Large VLDL	1.417	1.390	1.444	6.87E-272	4.97E-271	1.132	1.114	1.151	1.74E-50	9.80E-50	0.977	0.947	1.008	1.42E-01	3.28E-01	1.254	1.210	1.299	8.36E-36	7.47E-35
Cholesterol in Large VLDL	1.337	1.309	1.365	3.52E-165	1.16E-164	1.140	1.122	1.159	2.72E-55	2.30E-54	0.977	0.948	1.008	1.42E-01	3.28E-01	1.215	1.171	1.261	6.36E-25	2.10E-24
Cholesteryl Esters in Large VLDL	1.252	1.226	1.279	2.31E-94	5.32E-94	1.142	1.123	1.161	1.32E-56	1.34E-55	0.982	0.952	1.012	2.30E-01	3.95E-01	1.176	1.133	1.221	3.47E-17	9.09E-17
Free	1	1.	1.	4.66	2.15	1	1.	1.	2	1	0	0.	1.	9.	2.	1	1.	1.	4	2

Cholesterol in Large VLDL	.399	371	427	E-240	E-239	.1344	116	153	.24E-51	.36E-50	.974	944	004	05E-02	50E-01	.243	199	288	.15E-32	.10E-31
Triglycerides in Large VLDL	1.386	1.361	1.411	2.43E-275	1.85E-274	1.100	1.083	1.118	3.68E-32	8.74E-32	0.959	0.930	0.989	8.16E-03	5.17E-02	1.225	1.184	1.267	5.19E-32	2.54E-31
Concentration of Medium VLDL Particles	1.152	1.128	1.177	2.61E-39	4.67E-39	1.123	1.106	1.141	4.05E-46	1.81E-45	0.978	0.949	1.007	1.34E-01	3.28E-01	1.117	1.077	1.159	4.01E-09	7.52E-09
Total Lipids in Medium VLDL	1.224	1.199	1.250	1.29E-81	2.93E-81	1.125	1.108	1.144	2.39E-47	1.14E-46	0.972	0.943	1.002	6.32E-02	2.14E-01	1.155	1.114	1.198	1.01E-14	2.52E-14
Phospholipids in Medium VLDL	1.140	1.116	1.165	7.59E-34	1.31E-33	1.125	1.107	1.143	2.96E-47	1.36E-46	0.983	0.954	1.012	2.42E-01	3.95E-01	1.115	1.074	1.157	8.37E-09	1.53E-08
Cholesterol in	0.9	0.91	0.96	2.60E-08	3.19E-08	1.0	1.07	1.11	3.3	7.0	0.9	0.96	1.01	5.18	6.71	1.0	0.96	1.03	9.7	9.8

Medium VLDL	39	9	0			97	9	4	8E-300	4E-300	91	2	9	E-01	E-01	01	4	9	9E-01	4E-01
Cholesteryl Esters in Medium VLDL	0.846	0.827	0.865	3.75E-49	7.40E-49	1.076	1.059	1.093	1.24E-19	1.94E-19	0.995	0.967	1.024	7.43E-01	8.49E-01	0.943	0.908	0.979	2.22E-03	3.07E-03
Free Cholesterol in Medium VLDL	1.074	1.052	1.098	5.76E-11	7.36E-11	1.117	1.100	1.135	1.99E-42	6.58E-42	0.985	0.957	1.014	3.11E-01	4.55E-01	1.078	1.039	1.119	6.62E-05	1.05E-04
Triglycerides in Medium VLDL	1.363	1.337	1.389	5.32E-221	2.38E-220	1.115	1.097	1.133	1.38E-40	4.28E-40	0.962	0.933	0.992	1.37E-02	7.69E-02	1.220	1.179	1.264	4.51E-29	1.85E-28
Concentration of Small VLDL Particles	1.303	1.277	1.330	7.81E-146	2.37E-145	1.147	1.128	1.165	4.19E-63	1.27E-61	0.996	0.967	1.027	8.06E-01	8.94E-01	1.219	1.176	1.264	2.32E-27	8.60E-27
Total Lipids in	1.29	1.266	1.319	9.80E-134	2.66E-133	1.14	1.127	1.163	3.80	7.22	0.99	0.965	1.025	7.18E	8.27E	1.21	1.168	1.256	3.75	1.27

Small VLDL	2					5			E-61	E-60	4			-01	-01	1			E-25	E-24
Phospholipids in Small VLDL	1.169	1.145	1.194	2.53E-47	4.69E-47	1.137	1.119	1.155	1.50E-55	1.34E-54	0.994	0.965	1.023	6.71E-01	7.95E-01	1.143	1.102	1.186	1.17E-12	2.74E-12
Cholesterol in Small VLDL	1.120	1.096	1.145	4.92E-25	7.79E-25	1.139	1.121	1.158	2.40E-57	3.30E-56	1.004	0.975	1.034	7.95E-01	8.88E-01	1.121	1.080	1.163	2.16E-09	4.21E-09
Cholesteryl Esters in Small VLDL	1.153	1.128	1.178	2.77E-38	4.90E-38	1.148	1.130	1.167	2.90E-63	1.10E-61	1.010	0.980	1.040	5.21E-01	6.71E-01	1.143	1.101	1.187	2.58E-12	5.85E-12
Free Cholesterol in Small VLDL	1.064	1.041	1.087	2.05E-08	2.53E-08	1.122	1.104	1.140	8.71E-46	3.78E-45	0.994	0.965	1.023	6.80E-01	7.95E-01	1.081	1.042	1.122	3.96E-05	6.40E-05
Triglycerides in Small VL	1.404	1.379	1.430	2.72E-293	2.43E-292	1.121	1.104	1.139	1.43E-4	5.87E-4	0.988	0.959	1.018	4.31E-01	5.90E-01	1.260	1.218	1.303	2.37E-4	3.27E-4

DL									5	5									1	0
Concentration of Very Small VLDL Particles	1.131	1.107	1.155	4.24E-30	7.01E-30	1.133	1.115	1.151	2.73E-53	1.98E-52	1.043	1.013	1.074	4.39E-03	3.67E-02	1.144	1.103	1.186	4.91E-13	1.17E-12
Total Lipids in Very Small VLDL	1.139	1.115	1.164	1.45E-33	2.48E-33	1.135	1.117	1.153	5.78E-54	4.62E-53	1.044	1.014	1.075	3.53E-03	3.16E-02	1.152	1.111	1.195	3.55E-14	8.70E-14
Phospholipids in Very Small VLDL	1.173	1.149	1.197	1.31E-51	2.65E-51	1.146	1.128	1.164	7.66E-64	3.88E-62	1.050	1.020	1.081	1.05E-03	1.14E-02	1.181	1.139	1.224	1.02E-19	3.04E-19
Cholesterol in Very Small VLDL	0.963	0.942	0.985	1.02E-03	1.15E-03	1.102	1.084	1.120	2.00E-31	4.41E-31	1.039	1.009	1.070	9.68E-03	5.88E-02	1.038	0.999	1.078	5.79E-02	7.15E-02
Cholesteryl Esters in Very	0.913	0.892	0.934	4.00E-15	5.63E-15	1.087	1.070	1.105	1.18E-	2.02E-	1.037	1.007	1.068	1.43E-0	7.77E-0	1.002	0.964	1.041	9.25E-	9.56E-

Small VLDL									23	23				2	2				01	01
Free Cholesterol in Very Small VLDL	1.083	1.060	1.107	3.09E-13	4.23E-13	1.130	1.112	1.148	1.51E-50	8.83E-50	1.042	1.013	1.073	5.08E-03	3.67E-02	1.119	1.078	1.161	2.73E-09	5.19E-09
Triglycerides in Very Small VLDL	1.375	1.352	1.398	1.56E-306	1.58E-305	1.135	1.118	1.152	2.42E-59	3.68E-58	1.032	1.002	1.063	3.41E-02	1.36E-01	1.272	1.233	1.313	1.96E-51	9.93E-50
Concentration of IDL Particles	0.931	0.911	0.952	2.02E-10	2.54E-10	1.085	1.068	1.103	7.43E-24	1.28E-23	1.013	0.984	1.042	3.90E-01	5.41E-01	1.000	0.964	1.038	9.83E-01	9.84E-01
Total Lipids in IDL	0.895	0.875	0.916	9.85E-22	1.50E-21	1.066	1.049	1.084	1.61E-14	2.19E-14	1.011	0.982	1.041	4.62E-01	6.21E-01	0.976	0.939	1.014	2.18E-01	2.47E-01
Phospholipids in IDL	0.906	0.886	0.927	1.24E-17	1.83E-17	1.081	1.063	1.099	1.14E-	1.81E-	1.018	0.988	1.048	2.44E-0	3.95E-0	0.994	0.956	1.032	7.40E-	7.86E-

									20	20				1	1				01	01
Cholesterol in IDL	0.843	0.824	0.862	1.73E-49	3.46E-49	1.045	1.028	1.063	1.19E-07	1.46E-07	1.003	0.974	1.033	8.25E-01	9.01E-01	0.934	0.899	0.970	4.86E-04	7.10E-04
Cholesteryl Esters in IDL	0.845	0.827	0.865	1.40E-47	2.66E-47	1.044	1.027	1.062	2.56E-07	3.09E-07	1.002	0.973	1.032	9.05E-01	9.24E-01	0.935	0.899	0.971	5.94E-04	8.59E-04
Free Cholesterol in IDL	0.839	0.820	0.858	2.86E-52	5.87E-52	1.047	1.030	1.065	2.90E-08	3.58E-08	1.008	0.979	1.037	6.14E-01	7.53E-01	0.933	0.898	0.970	3.93E-04	5.80E-04
Triglycerides in IDL	1.343	1.320	1.366	1.30E-256	7.90E-256	1.140	1.122	1.157	4.37E-64	3.32E-62	1.049	1.019	1.079	1.30E-03	1.30E-02	1.263	1.224	1.303	3.66E-48	7.95E-47
Concentration of Large LDL Particles	0.989	0.968	1.010	2.99E-01	3.07E-01	1.109	1.092	1.127	1.96E-38	5.42E-38	0.994	0.965	1.023	6.63E-01	7.94E-01	1.039	1.001	1.078	4.16E-02	5.27E-02
Tota	0	0.	0.	7.98	9.33	1	1.	1.	1	2	0	0.	1.	2.	4.	1	0.	1.	9	9

l Lipids in Large LDL	.957	936	978	E-05	E-05	.075	057	092	.48E-18	.25E-18	.985	956	014	98E-01	41E-01	.001	964	039	.63E-01	.84E-01
Phospholipids in Large LDL	0.928	0.907	0.948	1.99 E-11	2.59 E-11	1.069	1.052	1.087	2.56E-16	3.67E-16	0.985	0.957	1.014	2.99E-01	4.41E-01	0.979	0.943	1.017	2.71E-01	3.05E-01
Cholesterol in Large LDL	0.928	0.907	0.948	2.54 E-11	3.27 E-11	1.066	1.049	1.083	8.33E-15	1.14E-14	0.980	0.952	1.009	1.82E-01	3.62E-01	0.980	0.944	1.018	3.03E-01	3.39E-01
Cholesteryl Esters in Large LDL	0.959	0.938	0.980	1.73 E-04	2.01 E-04	1.072	1.055	1.089	2.42E-17	3.57E-17	0.979	0.951	1.008	1.49E-01	3.39E-01	1.000	0.963	1.038	9.84E-01	9.84E-01
Free Cholesterol in Large LDL	0.848	0.829	0.867	1.28 E-47	2.46 E-47	1.047	1.030	1.064	2.16E-08	2.69E-08	0.985	0.957	1.014	3.17E-01	4.55E-01	0.930	0.895	0.966	1.67E-04	2.51E-04
Triglycerides in Larg	1.350	1.328	1.372	2.62 E-282	2.10 E-281	1.139	1.122	1.156	5.04E	7.66E	1.041	1.011	1.071	6.97E-	4.82E-	1.263	1.225	1.302	3.60E	1.09E

e LDL									-65	-63				03	02				-50	-48
Concentration of Medium LDL Particles	1.078	1.055	1.101	6.74E-12	8.91E-12	1.100	1.083	1.118	6.09E-32	1.38E-31	0.982	0.954	1.011	2.26E-01	3.95E-01	1.073	1.034	1.113	1.95E-04	2.90E-04
Total Lipids in Medium LDL	1.076	1.053	1.100	1.95E-11	2.56E-11	1.100	1.082	1.117	2.87E-31	6.23E-31	0.972	0.944	1.001	5.66E-02	2.00E-01	1.067	1.028	1.107	6.64E-04	9.53E-04
Phospholipids in Medium LDL	1.059	1.036	1.082	1.91E-07	2.30E-07	1.099	1.081	1.117	8.58E-31	1.84E-30	0.974	0.946	1.003	7.88E-02	2.35E-01	1.058	1.019	1.098	2.98E-03	4.05E-03
Cholesterol in Medium LDL	1.045	1.023	1.068	5.43E-05	6.40E-05	1.092	1.075	1.110	2.99E-27	6.06E-27	0.968	0.940	0.997	3.11E-02	1.35E-01	1.045	1.007	1.085	1.96E-02	2.52E-02
Cholesteryl Esters in Medium LDL	1.095	1.072	1.119	1.08E-16	1.56E-16	1.101	1.084	1.119	5.52E-32	1.27E-31	0.967	0.939	0.996	2.74E-02	1.23E-01	1.074	1.035	1.115	1.59E-04	2.42E-04

Free Cholesterol in Medium LDL	0.917	0.897	0.937	5.08E-15	7.08E-15	1.062	1.046	1.079	1.07E-13	1.41E-13	0.973	0.945	1.001	6.19E-02	2.14E-01	0.967	0.932	1.004	8.14E-02	9.98E-02
Triglycerides in Medium LDL	1.370	1.349	1.392	0.00E+00	0.00E+00	1.128	1.112	1.145	2.82E-57	3.30E-56	1.018	0.988	1.048	2.44E-01	3.95E-01	1.259	1.222	1.298	1.30E-51	9.88E-50
Concentration of Small LDL Particles	1.107	1.084	1.131	2.50E-21	3.76E-21	1.114	1.097	1.132	4.35E-41	1.38E-40	0.991	0.963	1.020	5.53E-01	6.83E-01	1.097	1.058	1.138	5.96E-07	1.05E-06
Total Lipids in Small LDL	1.079	1.056	1.102	1.99E-12	2.65E-12	1.107	1.090	1.125	1.10E-36	2.88E-36	0.980	0.952	1.009	1.70E-01	3.62E-01	1.080	1.041	1.120	3.37E-05	5.57E-05
Phospholipids in Small LDL	1.055	1.033	1.077	5.72E-07	6.85E-07	1.108	1.091	1.125	2.51E-37	5.69E-37	0.991	0.963	1.020	5.39E-01	6.77E-01	1.082	1.044	1.122	1.79E-05	3.02E-05
Cholesterol in	1.0	1.01	1.05	2.35E-03	2.61E-03	1.0	1.08	1.11	2.5	5.4	0.9	0.94	1.00	7.65	2.35	1.0	1.00	1.08	1.7	2.2

Small LDL	34	2	6			97	0	5	6E-30	0E-30	74	6	3	E-02	E-01	46	8	5	4E-02	6E-02
Cholesteryl Esters in Small LDL	1.088	1.065	1.111	6.04E-15	8.35E-15	1.106	1.089	1.124	1.03E-35	2.65E-35	0.973	0.945	1.002	7.13E-02	2.35E-01	1.076	1.037	1.116	9.08E-05	1.42E-04
Free Cholesterol in Small LDL	0.899	0.880	0.918	1.38E-22	2.12E-22	1.062	1.046	1.079	8.90E-14	1.19E-13	0.978	0.951	1.007	1.35E-01	3.28E-01	0.965	0.930	1.001	5.62E-02	7.01E-02
Triglycerides in Small LDL	1.376	1.354	1.398	0.00E+00	0.00E+00	1.114	1.097	1.131	2.85E-45	1.11E-44	1.000	0.971	1.030	1.00E+00	1.00E+00	1.247	1.210	1.285	7.63E-47	1.45E-45
Concentration of Very Large HDL Particles	0.695	0.672	0.719	2.40E-98	5.61E-98	0.936	0.917	0.956	7.37E-10	9.41E-10	1.043	1.008	1.079	1.57E-02	8.23E-02	0.870	0.825	0.918	3.78E-07	6.76E-07
Total Lipids in	0.64	0.622	0.667	5.19E-133	1.38E-132	0.91	0.899	0.938	4.26	5.89	1.04	1.012	1.083	7.96E	5.17E	0.83	0.790	0.882	1.46	3.04

Very Large HDL	4					8			E-15	E-15	7			-03	-02	5			E-10	E-10
Phospholipids in Very Large HDL	0.652	0.630	0.676	1.13E-125	2.96E-125	0.912	0.892	0.932	3.00E-17	4.38E-17	1.050	1.015	1.086	5.00E-03	3.67E-02	0.838	0.793	0.885	3.14E-10	6.36E-10
Cholesterol in Very Large HDL	0.599	0.578	0.620	3.16E-180	1.14E-179	0.915	0.896	0.935	3.18E-16	4.52E-16	1.040	1.005	1.076	2.37E-02	1.16E-01	0.801	0.758	0.847	4.06E-15	1.03E-14
Cholesteryl Esters in Very Large HDL	0.574	0.553	0.595	1.46E-198	5.55E-198	0.902	0.883	0.922	6.69E-21	1.07E-20	1.032	0.997	1.068	7.26E-02	2.35E-01	0.777	0.734	0.823	2.81E-18	7.63E-18
Free Cholesterol in Very Large HDL	0.718	0.697	0.740	1.10E-102	2.65E-102	0.967	0.949	0.986	8.85E-04	1.00E-03	1.065	1.031	1.100	1.28E-04	3.23E-03	0.899	0.856	0.944	2.06E-05	3.44E-05
Triglycerides	1.2	1.25	1.30	3.38E-178	1.19E-177	1.0	1.07	1.10	1.2	2.5	1.0	1.00	1.06	2.40	1.16	1.2	1.16	1.24	3.8	2.0

in Very Large HDL	80	9	2			86	0	3	5E-26	0E-26	33	4	3	E-02	E-01	06	9	4	2E-32	0E-31
Concentration of Large HDL Particles	0.592	0.571	0.614	2.62E-173	9.05E-173	0.856	0.837	0.875	5.99E-43	2.07E-42	1.009	0.974	1.046	6.21E-01	7.55E-01	0.745	0.703	0.790	3.52E-23	1.14E-22
Total Lipids in Large HDL	0.616	0.594	0.638	8.47E-156	2.68E-155	0.854	0.835	0.873	2.00E-44	7.41E-44	1.011	0.976	1.048	5.34E-01	6.77E-01	0.757	0.715	0.802	1.65E-21	5.02E-21
Phospholipids in Large HDL	0.669	0.646	0.692	3.02E-120	7.78E-120	0.857	0.838	0.876	6.24E-44	2.26E-43	1.011	0.976	1.047	5.34E-01	6.77E-01	0.785	0.742	0.829	8.63E-18	2.30E-17
Cholesterol in Large HDL	0.542	0.522	0.563	8.84E-221	3.73E-220	0.846	0.828	0.865	1.41E-48	6.91E-48	1.008	0.972	1.044	6.77E-01	7.95E-01	0.712	0.672	0.756	3.06E-29	1.29E-28
Cholesteryl Este	0.52	0.505	0.545	3.91E-241	1.98E-240	0.84	0.822	0.860	2.67	1.69	1.00	0.968	1.040	8.48E	9.01E	0.69	0.657	0.740	1.63	8.85

rs in Large HDL	4					1			E-52	E-51	3			-01	-01	7			E-32	E-32
Free Cholesterol in Large HDL	0.620	0.598	0.642	2.33E-150	7.23E-150	0.869	0.850	0.889	1.43E-35	3.62E-35	1.022	0.986	1.058	2.35E-01	3.95E-01	0.774	0.731	0.820	1.76E-18	4.86E-18
Triglycerides in Large HDL	1.202	1.178	1.226	3.77E-73	8.30E-73	1.036	1.019	1.053	3.15E-05	3.71E-05	1.039	1.009	1.070	1.11E-02	6.51E-02	1.146	1.105	1.188	9.25E-14	2.23E-13
Phospholipids in Medium HDL	0.971	0.948	0.995	1.81E-02	1.94E-02	0.907	0.891	0.924	1.96E-25	3.72E-25	0.966	0.935	0.997	3.22E-02	1.36E-01	0.921	0.883	0.961	1.55E-04	2.38E-04
Triglycerides in Medium HDL	1.344	1.319	1.368	7.14E-221	3.10E-220	1.075	1.058	1.092	1.07E-18	1.64E-18	1.013	0.983	1.043	3.91E-01	5.41E-01	1.209	1.169	1.251	4.55E-28	1.82E-27
Concentration of Small HD	1.022	0.999	1.045	5.79E-02	6.11E-02	0.979	0.963	0.995	1.21E-0	1.33E-0	0.924	0.897	0.953	3.18E-07	1.81E-05	0.949	0.913	0.987	9.01E-0	1.18E-0

L Particles									2	2									3	2
Total Lipids in Small HDL	1.127	1.103	1.152	2.24E-27	3.62E-27	0.987	0.971	1.004	1.34E-01	1.43E-01	0.937	0.909	0.966	2.66E-05	8.09E-04	1.012	0.973	1.052	5.52E-01	5.95E-01
Phospholipids in Small HDL	1.141	1.116	1.165	4.08E-33	6.89E-33	0.980	0.964	0.997	2.14E-02	2.34E-02	0.943	0.915	0.972	1.53E-04	3.32E-03	1.019	0.981	1.059	3.30E-01	3.64E-01
Cholesterol in Small HDL	0.978	0.957	1.000	5.36E-02	5.70E-02	0.964	0.948	0.980	1.90E-05	2.26E-05	0.925	0.898	0.953	3.57E-07	1.81E-05	0.921	0.886	0.958	4.25E-05	6.80E-05
Cholesteryl Esters in Small HDL	0.937	0.916	0.958	1.35E-08	1.68E-08	0.953	0.937	0.969	1.14E-08	1.43E-08	0.918	0.891	0.946	2.12E-08	3.22E-06	0.890	0.855	0.925	4.98E-09	9.23E-09
Free Cholesterol in Small HDL	1.110	1.086	1.134	7.05E-21	1.05E-20	1.005	0.988	1.022	5.55E-01	5.70E-01	0.957	0.929	0.987	4.73E-03	3.67E-02	1.033	0.994	1.074	9.53E-02	1.16E-01

Triglycerides in Small HDL	1.474	1.447	1.501	0.00E+00	0.00E+00	1.124	1.106	1.143	6.17E-45	2.34E-44	1.003	0.972	1.034	8.72E-01	9.21E-01	1.292	1.249	1.337	1.55E-48	3.93E-47

Abbreviations: HDL, high-density lipoprotein; IDL, intermediate-density lipoprotein; LDL, low-density lipoprotein; VLDL, very low-density lipoprotein.

Table S6 14 metabolites associated with all 3 CMDs and CMM.

Field_ID	Metabolites	Group	Subgroup	Association Direction with healthy sleep score
n_23427_0_0	Total Concentration of Lipoprotein Particles	Lipoprotein particle concentrations	NA	+
n_23730_0_0	Concentration of HDL Particles	Lipoprotein particle concentrations	NA	+
n_23731_0_0	Average Diameter for VLDL Particles	Lipoprotein particle sizes	NA	+
n_23743_0_0	Degree of Unsaturation	Fatty acids	NA	+
n_23778_0_0	Creatinine	Fluid balance	NA	+
n_23816_0_0	Concentration of Very Small VLDL Particles	Lipoprotein subclasses	Very small VLDL (average diameter 31.3 nm)	+
n_23817_0_0	Total Lipids in Very Small VLDL	Lipoprotein subclasses	Very small VLDL (average diameter 31.3 nm)	+
n_23818_0_0	Phospholipids in Very Small VLDL	Lipoprotein subclasses	Very small VLDL (average diameter 31.3 nm)	+

n_23821_0_0	Free Cholesterol in Very Small VLDL	Lipoprotein subclasses	Very small VLDL (average diameter 31.3 nm)	+
n_23829_0_0	Triglycerides in IDL	Lipoprotein subclasses	IDL (average diameter 28.6 nm)	+
n_23836_0_0	Triglycerides in Large LDL	Lipoprotein subclasses	Large LDL (average diameter 25.5 nm)	+
n_23853_0_0	Phospholipids in Very Large HDL	Lipoprotein subclasses	Very large HDL (average diameter 14.3 nm)	+
n_23856_0_0	Free Cholesterol in Very Large HDL	Lipoprotein subclasses	Very large HDL (average diameter 14.3 nm)	+
n_23876_0_0	Cholesteryl Esters in Small HDL	Lipoprotein subclasses	Small HDL (average diameter 8.7 nm)	+

Abbreviations: HDL, high-density lipoprotein; IDL, intermediate-density lipoprotein; LDL, low-density lipoprotein; VLDL, very low-density lipoprotein.

Table S7 Mediating effects of the circulating metabolites both associated with healthy sleep score and outcome.

**Metabolites**	**Mediating proportion, %**	**95%Low**	**95%Up**	***P* value**	***FDR***
**T2DM**					
Total Cholesterol	-1.51%	-2.18%	-0.98%	<0.001	<0.001
VLDL Cholesterol	4.24%	3.36%	5.66%	<0.001	<0.001
Clinical LDL Cholesterol	-1.52%	-2.21%	-1.03%	<0.001	<0.001
LDL Cholesterol	-0.69%	-1.27%	-0.22%	<0.001	<0.001
HDL Cholesterol	3.49%	2.24%	4.98%	<0.001	<0.001
Total Triglycerides	11.26%	9.33%	14.06%	<0.001	<0.001
Triglycerides in VLDL	11.36%	9.26%	14.06%	<0.001	<0.001
Triglycerides in	10.30%	8.63%	13.06%	<0.001	<0.001

LDL					
Triglycerides in HDL	7.12%	5.98%	8.75%	<0.001	<0.001
Total Phospholipids in Lipoprotein Particles	0.75%	0.29%	1.22%	<0.001	<0.001
Phospholipids in VLDL	8.76%	7.00%	11.18%	<0.001	<0.001
Total Esterified Cholesterol	-1.72%	-2.45%	-1.18%	<0.001	<0.001
Cholesteryl Esters in VLDL	2.14%	1.50%	2.96%	<0.001	<0.001
Cholesteryl Esters in HDL	4.14%	2.70%	5.76%	<0.001	<0.001
Total Free Cholesterol	-0.75%	-1.26%	-0.37%	<0.001	<0.001
Free Cholesterol in VLDL	7.25%	5.78%	8.78%	<0.001	<0.001
Free Cholesterol in LDL	-1.76%	-2.55%	-1.22%	<0.001	<0.001
Free Cholesterol in HDL	1.42%	0.74%	2.35%	<0.001	<0.001
Total Lipids in Lipoprotein Particles	2.83%	2.07%	3.82%	<0.001	<0.001
Total Lipids in VLDL	9.83%	8.13%	12.24%	<0.001	<0.001
Total Lipids in HDL	0.68%	0.03%	1.34%	0.04	0.04
Total Concentration of Lipoprotein Particles	-1.38%	-2.04%	-0.88%	<0.001	<0.001
Concentration of VLDL Particles	6.67%	5.36%	8.34%	<0.001	<0.001
Concentration of LDL Particles	0.55%	0.08%	1.02%	0.026666667	0.026855792
Concentration of HDL Particles	-1.09%	-1.70%	-0.59%	<0.001	<0.001
Average Diameter for VLDL Particles	12.15%	10.24%	14.68%	<0.001	<0.001
Average Diameter for LDL Particles	2.38%	1.50%	3.44%	<0.001	<0.001
Average Diameter for HDL Particles	9.24%	7.32%	12.04%	<0.001	<0.001
Phosphoglycerides	1.30%	0.81%	1.85%	<0.001	<0.001

Phosphatidylcholines	0.45%	0.15%	0.85%	0.006666667	0.006859904
Sphingomyelins	-1.63%	-2.45%	-1.13%	<0.001	<0.001
Apolipoprotein B	0.69%	0.21%	1.22%	<0.001	<0.001
Total Fatty Acids	7.90%	6.52%	9.84%	<0.001	<0.001
Degree of Unsaturation	7.89%	6.20%	9.82%	<0.001	<0.001
Omega-3 Fatty Acids	0.70%	0.40%	1.17%	<0.001	<0.001
Omega-6 Fatty Acids	0.73%	0.28%	1.36%	<0.001	<0.001
Polyunsaturated Fatty Acids	1.04%	0.50%	1.60%	<0.001	<0.001
Monounsaturated Fatty Acids	10.72%	8.94%	13.14%	<0.001	<0.001
Saturated Fatty Acids	9.17%	7.48%	10.95%	<0.001	<0.001
Alanine	1.54%	0.85%	2.39%	<0.001	<0.001
Glutamine	1.26%	0.69%	1.85%	<0.001	<0.001
Total Concentration of Branched-Chain Amino Acids (Leucine + Isoleucine + Valine)	1.47%	0.72%	2.38%	<0.001	<0.001
Isoleucine	0.67%	0.13%	1.21%	0.013333333	0.013621103
Leucine	1.08%	0.53%	1.63%	<0.001	<0.001
Valine	2.00%	1.14%	2.89%	<0.001	<0.001
Tyrosine	2.40%	1.67%	3.33%	<0.001	<0.001
Glucose	1.86%	0.95%	2.88%	<0.001	<0.001
Lactate	0.68%	0.24%	1.19%	0.006666667	0.006859904
Citrate	-0.78%	-1.33%	-0.24%	0.006666667	0.006859904
Acetate	1.05%	0.56%	1.71%	<0.001	<0.001
Creatinine	0.53%	0.31%	0.82%	<0.001	<0.001
Glycoprotein Acetyls	10.97%	9.16%	13.29%	<0.001	<0.001
Concentration of Chylomicrons and Extremely Large VLDL Particles	10.21%	8.27%	12.65%	<0.001	<0.001
Total Lipids in Chylomicrons and	9.89%	8.05%	11.94%	<0.001	<0.001

Extremely Large VLDL					
Phospholipids in Chylomicrons and Extremely Large VLDL	10.16%	8.33%	12.64%	<0.001	<0.001
Cholesterol in Chylomicrons and Extremely Large VLDL	10.11%	8.23%	12.45%	<0.001	<0.001
Cholesteryl Esters in Chylomicrons and Extremely Large VLDL	9.89%	8.25%	12.12%	<0.001	<0.001
Free Cholesterol in Chylomicrons and Extremely Large VLDL	10.12%	8.11%	12.35%	<0.001	<0.001
Triglycerides in Chylomicrons and Extremely Large VLDL	9.02%	7.37%	11.28%	<0.001	<0.001
Concentration of Very Large VLDL Particles	11.72%	9.88%	14.70%	<0.001	<0.001
Total Lipids in Very Large VLDL	11.71%	9.61%	14.62%	<0.001	<0.001
Phospholipids in Very Large VLDL	11.24%	9.37%	13.91%	<0.001	<0.001
Cholesterol in Very Large VLDL	9.63%	8.00%	12.05%	<0.001	<0.001
Cholesteryl Esters in Very Large VLDL	7.58%	6.03%	9.47%	<0.001	<0.001
Free Cholesterol in Very Large VLDL	10.79%	9.13%	13.38%	<0.001	<0.001
Triglycerides in Very Large VLDL	11.58%	9.60%	13.75%	<0.001	<0.001
Concentration of Large VLDL Particles	11.07%	9.05%	13.57%	<0.001	<0.001
Total Lipids in Large VLDL	10.69%	9.10%	13.36%	<0.001	<0.001
Phospholipids in	11.48%	9.55%	13.97%	<0.001	<0.001

Large VLDL					
Cholesterol in Large VLDL	9.00%	7.46%	11.02%	<0.001	<0.001
Cholesteryl Esters in Large VLDL	6.53%	5.32%	8.39%	<0.001	<0.001
Free Cholesterol in Large VLDL	11.15%	9.24%	13.48%	<0.001	<0.001
Triglycerides in Large VLDL	10.39%	8.51%	12.51%	<0.001	<0.001
Concentration of Medium VLDL Particles	3.91%	3.09%	4.95%	<0.001	<0.001
Total Lipids in Medium VLDL	6.01%	4.95%	7.58%	<0.001	<0.001
Phospholipids in Medium VLDL	3.53%	2.76%	4.56%	<0.001	<0.001
Cholesterol in Medium VLDL	-1.14%	-1.64%	-0.65%	<0.001	<0.001
Cholesteryl Esters in Medium VLDL	-1.78%	-2.59%	-1.17%	<0.001	<0.001
Free Cholesterol in Medium VLDL	1.72%	1.11%	2.42%	<0.001	<0.001
Triglycerides in Medium VLDL	9.98%	8.22%	12.74%	<0.001	<0.001
Concentration of Small VLDL Particles	8.05%	6.52%	9.77%	<0.001	<0.001
Total Lipids in Small VLDL	7.71%	6.31%	9.52%	<0.001	<0.001
Phospholipids in Small VLDL	4.23%	3.38%	5.30%	<0.001	<0.001
Cholesterol in Small VLDL	2.83%	2.13%	3.72%	<0.001	<0.001
Cholesteryl Esters in Small VLDL	3.59%	2.79%	4.64%	<0.001	<0.001
Free Cholesterol in Small VLDL	1.41%	0.85%	2.08%	<0.001	<0.001
Triglycerides in Small VLDL	10.77%	8.99%	13.16%	<0.001	<0.001
Concentration of Very Small VLDL Particles	2.35%	1.80%	3.07%	<0.001	<0.001
Total Lipids in Very Small VLDL	2.53%	1.93%	3.38%	<0.001	<0.001

Phospholipids in Very Small VLDL	3.23%	2.49%	4.18%	<0.001	<0.001
Cholesterol in Very Small VLDL	-0.43%	-0.79%	-0.19%	<0.001	<0.001
Cholesteryl Esters in Very Small VLDL	-0.78%	-1.18%	-0.43%	<0.001	<0.001
Free Cholesterol in Very Small VLDL	1.40%	0.93%	2.02%	<0.001	<0.001
Triglycerides in Very Small VLDL	9.50%	7.99%	11.66%	<0.001	<0.001
Concentration of IDL Particles	-0.97%	-1.48%	-0.51%	<0.001	<0.001
Total Lipids in IDL	-1.22%	-1.70%	-0.79%	<0.001	<0.001
Total Lipids in LDL	-1.09%	-1.64%	-0.71%	<0.001	<0.001
Cholesterol in IDL	-1.34%	-2.06%	-0.77%	<0.001	<0.001
Cholesteryl Esters in IDL	-1.38%	-2.12%	-0.81%	<0.001	<0.001
Free Cholesterol in IDL	-1.26%	-2.01%	-0.58%	<0.001	<0.001
Triglycerides in IDL	8.54%	7.13%	10.44%	<0.001	<0.001
Total Lipids in Large LDL	-0.84%	-1.34%	-0.40%	<0.001	<0.001
Phospholipids in Large LDL	-1.39%	-1.92%	-0.92%	<0.001	<0.001
Cholesterol in Large LDL	-1.25%	-1.95%	-0.80%	<0.001	<0.001
Cholesteryl Esters in Large LDL	-0.83%	-1.31%	-0.41%	<0.001	<0.001
Free Cholesterol in Large LDL	-1.75%	-2.48%	-1.11%	<0.001	<0.001
Triglycerides in Large LDL	9.61%	8.20%	11.52%	<0.001	<0.001
Concentration of Medium LDL Particles	1.76%	1.19%	2.70%	<0.001	<0.001
Total Lipids in Medium LDL	1.89%	1.30%	2.67%	<0.001	<0.001
Phospholipids in Medium LDL	1.40%	0.82%	2.09%	<0.001	<0.001
Cholesterol in Medium LDL	1.03%	0.46%	1.71%	<0.001	<0.001
Cholesteryl Esters	2.47%	1.78%	3.31%	<0.001	<0.001

in Medium LDL					
Free Cholesterol in Medium LDL	-1.45%	-2.06%	-1.01%	<0.001	<0.001
Triglycerides in Medium LDL	10.79%	8.64%	13.20%	<0.001	<0.001
Concentration of Small LDL Particles	2.37%	1.74%	3.29%	<0.001	<0.001
Total Lipids in Small LDL	1.84%	1.23%	2.45%	<0.001	<0.001
Phospholipids in Small LDL	1.11%	0.61%	1.64%	<0.001	<0.001
Cholesterol in Small LDL	0.76%	0.21%	1.32%	0.006666667	0.006859904
Cholesteryl Esters in Small LDL	2.17%	1.57%	2.92%	<0.001	<0.001
Free Cholesterol in Small LDL	-1.32%	-1.92%	-0.85%	<0.001	<0.001
Triglycerides in Small LDL	10.82%	8.95%	13.74%	<0.001	<0.001
Concentration of Very Large HDL Particles	5.89%	4.55%	7.78%	<0.001	<0.001
Total Lipids in Very Large HDL	8.54%	6.87%	10.83%	<0.001	<0.001
Phospholipids in Very Large HDL	8.79%	6.90%	10.78%	<0.001	<0.001
Cholesterol in Very Large HDL	10.14%	8.03%	13.02%	<0.001	<0.001
Cholesteryl Esters in Very Large HDL	10.82%	8.92%	13.30%	<0.001	<0.001
Free Cholesterol in Very Large HDL	6.35%	5.13%	8.02%	<0.001	<0.001
Triglycerides in Very Large HDL	4.38%	3.39%	5.69%	<0.001	<0.001
Concentration of Large HDL Particles	8.83%	7.10%	11.38%	<0.001	<0.001
Total Lipids in Large HDL	8.21%	6.33%	10.52%	<0.001	<0.001
Phospholipids in Large HDL	6.38%	4.79%	8.18%	<0.001	<0.001
Cholesterol in Large HDL	11.51%	9.30%	14.21%	<0.001	<0.001
Cholesteryl Esters	12.33%	9.90%	15.13%	<0.001	<0.001

in Large HDL					
Free Cholesterol in Large HDL	8.27%	6.56%	10.38%	<0.001	<0.001
Triglycerides in Large HDL	1.11%	0.54%	1.80%	<0.001	<0.001
Phospholipids in Medium HDL	-0.15%	-0.39%	-0.02%	0.026666667	0.026855792
Triglycerides in Medium HDL	6.94%	5.53%	8.74%	<0.001	<0.001
Total Lipids in Small HDL	3.18%	2.40%	4.26%	<0.001	<0.001
Phospholipids in Small HDL	3.25%	2.46%	4.26%	<0.001	<0.001
Cholesteryl Esters in Small HDL	-1.40%	-2.03%	-0.94%	<0.001	<0.001
Free Cholesterol in Small HDL	2.45%	1.73%	3.28%	<0.001	<0.001
Triglycerides in Small HDL	12.26%	10.24%	15.37%	<0.001	<0.001
**CHD**					
Total Cholesterol	0.95%	0.49%	1.46%	<0.001	<0.001
Total Cholesterol Minus HDL-C	2.37%	1.75%	3.10%	<0.001	<0.001
Remnant Cholesterol (Non-HDL, Non-LDL -Cholesterol)	2.70%	2.01%	3.50%	<0.001	<0.001
VLDL Cholesterol	4.69%	3.60%	6.00%	<0.001	<0.001
Clinical LDL Cholesterol	1.58%	1.06%	2.18%	<0.001	<0.001
LDL Cholesterol	2.00%	1.44%	2.71%	<0.001	<0.001
HDL Cholesterol	1.90%	1.20%	2.85%	<0.001	<0.001
Total Triglycerides	4.60%	3.62%	5.96%	<0.001	<0.001
Triglycerides in VLDL	4.38%	3.22%	5.57%	<0.001	<0.001
Triglycerides in LDL	5.61%	4.49%	7.09%	<0.001	<0.001
Triglycerides in HDL	2.57%	1.95%	3.66%	<0.001	<0.001
Total Phospholipids in Lipoprotein Particles	0.78%	0.40%	1.25%	<0.001	<0.001
Phospholipids in VLDL	5.54%	4.44%	6.90%	<0.001	<0.001

Phospholipids in LDL	2.29%	1.74%	3.03%	<0.001	<0.001
Total Esterified Cholesterol	0.69%	0.34%	1.14%	<0.001	<0.001
Cholesteryl Esters in VLDL	3.97%	3.03%	5.10%	<0.001	<0.001
Cholesteryl Esters in LDL	2.43%	1.80%	3.28%	<0.001	<0.001
Cholesteryl Esters in HDL	2.13%	1.49%	3.04%	<0.001	<0.001
Total Free Cholesterol	1.60%	1.09%	2.28%	<0.001	<0.001
Free Cholesterol in VLDL	5.31%	4.22%	6.80%	<0.001	<0.001
Free Cholesterol in LDL	0.86%	0.56%	1.36%	<0.001	<0.001
Free Cholesterol in HDL	0.86%	0.45%	1.41%	<0.001	<0.001
Total Lipids in Lipoprotein Particles	2.44%	1.68%	3.28%	<0.001	<0.001
Total Lipids in VLDL	5.16%	4.10%	6.54%	<0.001	<0.001
Total Lipids in LDL	2.38%	1.65%	3.20%	<0.001	<0.001
Total Lipids in HDL	0.58%	0.08%	1.20%	0.013333333	0.013625304
Total Concentration of Lipoprotein Particles	-1.04%	-1.69%	-0.67%	<0.001	<0.001
Concentration of VLDL Particles	5.30%	4.22%	6.79%	<0.001	<0.001
Concentration of LDL Particles	3.18%	2.36%	4.17%	<0.001	<0.001
Concentration of HDL Particles	-1.01%	-1.57%	-0.59%	<0.001	<0.001
Average Diameter for VLDL Particles	2.84%	2.08%	4.03%	<0.001	<0.001
Average Diameter for HDL Particles	4.37%	3.40%	5.83%	<0.001	<0.001
Sphingomyelins	0.39%	0.09%	0.76%	0.006666667	0.006862745
Apolipoprotein B	3.25%	2.57%	4.23%	<0.001	<0.001
Total Fatty Acids	3.68%	2.86%	4.83%	<0.001	<0.001
Degree of	2.27%	1.53%	2.95%	<0.001	<0.001

Unsaturation					
Omega-6 Fatty Acids	1.70%	1.10%	2.35%	<0.001	<0.001
Polyunsaturated Fatty Acids	1.41%	0.90%	2.16%	<0.001	<0.001
Monounsaturated Fatty Acids	4.94%	3.88%	6.33%	<0.001	<0.001
Saturated Fatty Acids	3.57%	2.74%	4.60%	<0.001	<0.001
Linoleic Acid	1.25%	0.76%	1.82%	<0.001	<0.001
Total Concentration of Branched-Chain Amino Acids (Leucine + Isoleucine + Valine)	0.30%	0.09%	0.56%	<0.001	<0.001
Isoleucine	0.20%	0.04%	0.44%	0.02	0.020289855
Leucine	0.23%	0.06%	0.47%	<0.001	<0.001
Valine	0.30%	0.11%	0.57%	<0.001	<0.001
Tyrosine	0.43%	0.15%	0.81%	<0.001	<0.001
Citrate	0.53%	0.04%	1.15%	0.033333333	0.033333333
Creatinine	0.80%	0.49%	1.20%	<0.001	<0.001
Glycoprotein Acetyls	6.67%	5.30%	8.19%	<0.001	<0.001
Concentration of Chylomicrons and Extremely Large VLDL Particles	3.29%	2.51%	4.41%	<0.001	<0.001
Total Lipids in Chylomicrons and Extremely Large VLDL	3.06%	2.16%	4.06%	<0.001	<0.001
Phospholipids in Chylomicrons and Extremely Large VLDL	3.22%	2.36%	4.32%	<0.001	<0.001
Cholesterol in Chylomicrons and Extremely Large VLDL	3.82%	2.90%	5.04%	<0.001	<0.001
Cholesteryl Esters in Chylomicrons and Extremely	4.15%	3.21%	5.28%	<0.001	<0.001

Large VLDL					
Free Cholesterol in Chylomicrons and Extremely Large VLDL	3.35%	2.50%	4.46%	<0.001	<0.001
Triglycerides in Chylomicrons and Extremely Large VLDL	2.59%	1.83%	3.54%	<0.001	<0.001
Concentration of Very Large VLDL Particles	4.93%	3.98%	6.43%	<0.001	<0.001
Total Lipids in Very Large VLDL	4.72%	3.68%	6.12%	<0.001	<0.001
Phospholipids in Very Large VLDL	4.96%	3.91%	6.43%	<0.001	<0.001
Cholesterol in Very Large VLDL	5.53%	4.49%	6.99%	<0.001	<0.001
Cholesteryl Esters in Very Large VLDL	5.66%	4.39%	7.22%	<0.001	<0.001
Free Cholesterol in Very Large VLDL	5.12%	3.91%	6.78%	<0.001	<0.001
Triglycerides in Very Large VLDL	4.05%	3.19%	5.43%	<0.001	<0.001
Concentration of Large VLDL Particles	5.13%	4.13%	6.45%	<0.001	<0.001
Total Lipids in Large VLDL	4.82%	3.78%	6.09%	<0.001	<0.001
Phospholipids in Large VLDL	5.53%	4.36%	7.02%	<0.001	<0.001
Cholesterol in Large VLDL	5.60%	4.49%	7.18%	<0.001	<0.001
Cholesteryl Esters in Large VLDL	5.28%	4.26%	6.95%	<0.001	<0.001
Free Cholesterol in Large VLDL	5.61%	4.42%	7.09%	<0.001	<0.001
Triglycerides in Large VLDL	3.99%	3.04%	5.21%	<0.001	<0.001
Concentration of Medium VLDL Particles	4.40%	3.41%	5.56%	<0.001	<0.001
Total Lipids in	4.78%	3.64%	5.99%	<0.001	<0.001

Medium VLDL					
Phospholipids in Medium VLDL	4.38%	3.44%	5.70%	<0.001	<0.001
Cholesterol in Medium VLDL	2.18%	1.61%	2.92%	<0.001	<0.001
Cholesteryl Esters in Medium VLDL	1.03%	0.62%	1.64%	<0.001	<0.001
Free Cholesterol in Medium VLDL	3.69%	2.90%	4.76%	<0.001	<0.001
Triglycerides in Medium VLDL	4.76%	3.74%	6.10%	<0.001	<0.001
Concentration of Small VLDL Particles	5.79%	4.66%	7.31%	<0.001	<0.001
Total Lipids in Small VLDL	5.49%	4.45%	7.03%	<0.001	<0.001
Phospholipids in Small VLDL	4.83%	3.67%	6.03%	<0.001	<0.001
Cholesterol in Small VLDL	4.54%	3.54%	5.80%	<0.001	<0.001
Cholesteryl Esters in Small VLDL	4.88%	3.91%	6.44%	<0.001	<0.001
Free Cholesterol in Small VLDL	3.77%	2.83%	4.84%	<0.001	<0.001
Triglycerides in Small VLDL	4.77%	3.71%	6.29%	<0.001	<0.001
Concentration of Very Small VLDL Particles	3.30%	2.49%	4.32%	<0.001	<0.001
Total Lipids in Very Small VLDL	3.46%	2.65%	4.52%	<0.001	<0.001
Phospholipids in Very Small VLDL	3.82%	2.96%	4.97%	<0.001	<0.001
Cholesterol in Very Small VLDL	1.50%	1.01%	2.22%	<0.001	<0.001
Cholesteryl Esters in Very Small VLDL	0.98%	0.56%	1.51%	<0.001	<0.001
Free Cholesterol in Very Small VLDL	2.98%	2.19%	3.90%	<0.001	<0.001
Triglycerides in Very Small VLDL	4.98%	3.84%	6.26%	<0.001	<0.001
Concentration of IDL Particles	1.47%	1.01%	2.12%	<0.001	<0.001

Total Lipids in IDL	0.95%	0.62%	1.43%	<0.001	<0.001
Phospholipids in IDL	1.15%	0.75%	1.62%	<0.001	<0.001
Cholesterol in IDL	0.47%	0.25%	0.81%	<0.001	<0.001
Cholesteryl Esters in IDL	0.45%	0.24%	0.81%	<0.001	<0.001
Free Cholesterol in IDL	0.43%	0.20%	0.73%	<0.001	<0.001
Triglycerides in IDL	5.14%	4.10%	6.68%	<0.001	<0.001
Concentration of Large LDL Particles	2.81%	2.10%	3.75%	<0.001	<0.001
Total Lipids in Large LDL	1.79%	1.23%	2.48%	<0.001	<0.001
Phospholipids in Large LDL	1.57%	1.13%	2.24%	<0.001	<0.001
Cholesterol in Large LDL	1.42%	0.96%	2.02%	<0.001	<0.001
Cholesteryl Esters in Large LDL	1.76%	1.21%	2.45%	<0.001	<0.001
Free Cholesterol in Large LDL	0.65%	0.34%	1.04%	<0.001	<0.001
Triglycerides in Large LDL	5.69%	4.54%	7.23%	<0.001	<0.001
Concentration of Medium LDL Particles	3.16%	2.47%	4.17%	<0.001	<0.001
Total Lipids in Medium LDL	3.40%	2.63%	4.44%	<0.001	<0.001
Phospholipids in Medium LDL	3.27%	2.43%	4.31%	<0.001	<0.001
Cholesterol in Medium LDL	3.07%	2.25%	4.01%	<0.001	<0.001
Cholesteryl Esters in Medium LDL	3.70%	2.69%	4.81%	<0.001	<0.001
Free Cholesterol in Medium LDL	1.37%	0.87%	1.92%	<0.001	<0.001
Triglycerides in Medium LDL	5.62%	4.58%	7.11%	<0.001	<0.001
Concentration of Small LDL Particles	3.59%	2.75%	4.62%	<0.001	<0.001
Total Lipids in Small LDL	3.49%	2.69%	4.42%	<0.001	<0.001
Phospholipids in	2.89%	2.21%	3.80%	<0.001	<0.001

Small LDL					
Cholesterol in Small LDL	3.03%	2.27%	4.00%	<0.001	<0.001
Cholesteryl Esters in Small LDL	3.78%	2.92%	4.86%	<0.001	<0.001
Free Cholesterol in Small LDL	1.02%	0.67%	1.48%	<0.001	<0.001
Triglycerides in Small LDL	4.80%	3.84%	6.21%	<0.001	<0.001
Concentration of Very Large HDL Particles	1.47%	0.93%	2.08%	<0.001	<0.001
Total Lipids in Very Large HDL	2.25%	1.62%	3.08%	<0.001	<0.001
Phospholipids in Very Large HDL	2.59%	1.90%	3.62%	<0.001	<0.001
Cholesterol in Very Large HDL	2.41%	1.74%	3.17%	<0.001	<0.001
Cholesteryl Esters in Very Large HDL	2.74%	2.03%	3.78%	<0.001	<0.001
Free Cholesterol in Very Large HDL	0.85%	0.26%	1.46%	<0.001	<0.001
Triglycerides in Very Large HDL	1.99%	1.33%	2.69%	<0.001	<0.001
Concentration of Large HDL Particles	3.61%	2.78%	4.72%	<0.001	<0.001
Total Lipids in Large HDL	3.70%	2.75%	4.68%	<0.001	<0.001
Phospholipids in Large HDL	3.42%	2.67%	4.53%	<0.001	<0.001
Cholesterol in Large HDL	4.31%	3.36%	5.52%	<0.001	<0.001
Cholesteryl Esters in Large HDL	4.56%	3.60%	5.77%	<0.001	<0.001
Free Cholesterol in Large HDL	3.32%	2.43%	4.49%	<0.001	<0.001
Triglycerides in Large HDL	0.27%	0.09%	0.54%	<0.001	<0.001
Phospholipids in Medium HDL	-0.79%	-1.27%	-0.35%	<0.001	<0.001
Triglycerides in Medium HDL	2.25%	1.63%	3.04%	<0.001	<0.001
Concentration of	-0.81%	-1.44%	-0.25%	<0.001	<0.001

Small HDL Particles					
Phospholipids in Small HDL	-0.82%	-1.29%	-0.14%	0.026666667	0.026858514
Cholesterol in Small HDL	-1.19%	-1.85%	-0.58%	<0.001	<0.001
Cholesteryl Esters in Small HDL	-1.46%	-2.09%	-0.90%	<0.001	<0.001
Triglycerides in Small HDL	5.05%	3.88%	6.55%	<0.001	<0.001
**Stroke**					
Total Concentration of Lipoprotein Particles	-1.48%	-3.49%	-0.60%	<0.001	<0.001
Concentration of HDL Particles	-1.02%	-2.51%	-0.38%	<0.001	<0.001
Average Diameter for VLDL Particles	-5.35%	-10.58%	-2.67%	<0.001	<0.001
Degree of Unsaturation	2.45%	1.02%	6.57%	<0.001	<0.001
Omega-6 Fatty Acids	-2.44%	-5.49%	-0.86%	<0.001	<0.001
Polyunsaturated Fatty Acids	-2.52%	-5.49%	-1.01%	<0.001	<0.001
Linoleic Acid	-1.92%	-4.71%	-0.65%	0.006666667	0.007719299
Creatinine	0.97%	0.34%	2.48%	<0.001	<0.001
Concentration of Very Small VLDL Particles	1.77%	0.34%	4.08%	0.026666667	0.026666667
Total Lipids in Very Small VLDL	1.98%	0.52%	4.28%	0.013333333	0.014666666
Phospholipids in Very Small VLDL	2.25%	0.77%	6.11%	<0.001	<0.001
Free Cholesterol in Very Small VLDL	1.68%	0.64%	3.93%	0.02	0.020952381
Triglycerides in IDL	3.07%	1.12%	7.24%	<0.001	<0.001
Triglycerides in Large LDL	2.87%	0.76%	7.35%	0.006666667	0.007719299
Phospholipids in Very Large HDL	-2.32%	-6.02%	-0.80%	0.006666667	0.007719299
Free Cholesterol in Very Large HDL	-2.85%	-6.87%	-1.20%	<0.001	<0.001

Concentration of Small HDL Particles	-4.69%	-9.87%	-2.43%	<0.001	<0.001
Total Lipids in Small HDL	-4.34%	-9.33%	-2.19%	<0.001	<0.001
Phospholipids in Small HDL	-3.41%	-8.61%	-1.46%	<0.001	<0.001
Cholesterol in Small HDL	-4.19%	-10.01%	-2.22%	<0.001	<0.001
Cholesteryl Esters in Small HDL	-4.12%	-8.54%	-2.29%	<0.001	<0.001
Free Cholesterol in Small HDL	-2.42%	-5.58%	-0.86%	<0.001	<0.001
**CMM**					
Remnant Cholesterol (Non-HDL, Non-LDL - Cholesterol)	0.83%	0.12%	1.58%	0.013333333	0.013678161
VLDL Cholesterol	3.31%	2.19%	4.89%	<0.001	<0.001
HDL Cholesterol	2.38%	1.36%	3.90%	<0.001	<0.001
Total Triglycerides	6.87%	4.99%	9.99%	<0.001	<0.001
Triglycerides in VLDL	6.48%	4.87%	9.20%	<0.001	<0.001
Triglycerides in LDL	7.12%	5.31%	9.72%	<0.001	<0.001
Triglycerides in HDL	4.41%	3.17%	6.53%	<0.001	<0.001
Phospholipids in VLDL	5.82%	4.21%	8.49%	<0.001	<0.001
Total Esterified Cholesterol	-0.75%	-1.41%	-0.25%	<0.001	<0.001
Cholesteryl Esters in VLDL	2.09%	1.19%	3.42%	<0.001	<0.001
Cholesteryl Esters in HDL	2.78%	1.80%	4.17%	<0.001	<0.001
Free Cholesterol in VLDL	4.85%	3.36%	6.86%	<0.001	<0.001
Free Cholesterol in LDL	-0.67%	-1.24%	-0.24%	0.006666667	0.007020649
Free Cholesterol in HDL	0.88%	0.40%	1.66%	<0.001	<0.001
Total Lipids in Lipoprotein Particles	1.87%	0.96%	3.07%	<0.001	<0.001

Total Lipids in VLDL	6.29%	4.51%	8.87%	<0.001	<0.001
Total Lipids in HDL	0.55%	0.08%	1.23%	0.026666667	0.026892656
Total Concentration of Lipoprotein Particles	-1.35%	-2.26%	-0.73%	<0.001	<0.001
Concentration of VLDL Particles	4.74%	3.41%	7.29%	<0.001	<0.001
Concentration of LDL Particles	1.16%	0.41%	2.17%	<0.001	<0.001
Concentration of HDL Particles	-1.13%	-1.94%	-0.56%	<0.001	<0.001
Average Diameter for VLDL Particles	5.50%	3.81%	7.67%	<0.001	<0.001
Average Diameter for LDL Particles	0.94%	0.49%	1.74%	<0.001	<0.001
Average Diameter for HDL Particles	4.68%	3.36%	6.98%	<0.001	<0.001
Sphingomyelins	-0.60%	-1.18%	-0.10%	0.02	0.02034188
Apolipoprotein B	1.24%	0.42%	2.22%	<0.001	<0.001
Total Fatty Acids	4.72%	3.19%	6.60%	<0.001	<0.001
Degree of Unsaturation	4.97%	3.54%	7.31%	<0.001	<0.001
Monounsaturated Fatty Acids	6.77%	4.85%	9.83%	<0.001	<0.001
Saturated Fatty Acids	5.53%	3.99%	8.16%	<0.001	<0.001
Alanine	0.88%	0.41%	1.58%	<0.001	<0.001
Glutamine	0.47%	0.15%	0.93%	0.006666667	0.007020649
Total Concentration of Branched-Chain Amino Acids (Leucine + Isoleucine + Valine)	0.66%	0.32%	1.14%	<0.001	<0.001
Isoleucine	0.35%	0.08%	0.74%	<0.001	<0.001
Leucine	0.39%	0.15%	0.81%	<0.001	<0.001
Valine	0.93%	0.45%	1.55%	<0.001	<0.001
Tyrosine	1.36%	0.83%	2.26%	<0.001	<0.001
Glucose	1.20%	0.57%	1.91%	<0.001	<0.001
Lactate	0.43%	0.14%	0.87%	<0.001	<0.001
Acetate	0.73%	0.27%	1.61%	<0.001	<0.001
Creatinine	0.66%	0.34%	1.06%	<0.001	<0.001

Glycoprotein Acetyls	7.68%	5.81%	11.13%	<0.001	<0.001
Concentration of Chylomicrons and Extremely Large VLDL Particles	5.86%	4.42%	8.35%	<0.001	<0.001
Total Lipids in Chylomicrons and Extremely Large VLDL	5.61%	4.00%	8.05%	<0.001	<0.001
Phospholipids in Chylomicrons and Extremely Large VLDL	5.73%	4.14%	8.13%	<0.001	<0.001
Cholesterol in Chylomicrons and Extremely Large VLDL	5.59%	4.01%	8.01%	<0.001	<0.001
Cholesteryl Esters in Chylomicrons and Extremely Large VLDL	5.60%	4.08%	8.03%	<0.001	<0.001
Free Cholesterol in Chylomicrons and Extremely Large VLDL	5.58%	3.99%	7.87%	<0.001	<0.001
Triglycerides in Chylomicrons and Extremely Large VLDL	5.08%	3.76%	7.64%	<0.001	<0.001
Concentration of Very Large VLDL Particles	7.02%	5.12%	9.86%	<0.001	<0.001
Total Lipids in Very Large VLDL	6.78%	5.05%	10.18%	<0.001	<0.001
Phospholipids in Very Large VLDL	6.64%	4.93%	9.33%	<0.001	<0.001
Cholesterol in Very Large VLDL	5.83%	4.20%	8.61%	<0.001	<0.001
Cholesteryl Esters in Very Large VLDL	4.81%	3.44%	7.22%	<0.001	<0.001
Free Cholesterol in Very Large VLDL	6.47%	4.70%	9.44%	<0.001	<0.001

Triglycerides in Very Large VLDL	6.55%	4.89%	9.13%	<0.001	<0.001
Concentration of Large VLDL Particles	6.67%	4.80%	9.79%	<0.001	<0.001
Total Lipids in Large VLDL	6.38%	4.75%	8.75%	<0.001	<0.001
Phospholipids in Large VLDL	6.72%	4.97%	9.90%	<0.001	<0.001
Cholesterol in Large VLDL	5.72%	4.07%	8.15%	<0.001	<0.001
Cholesteryl Esters in Large VLDL	4.43%	3.03%	6.31%	<0.001	<0.001
Free Cholesterol in Large VLDL	6.70%	4.75%	9.14%	<0.001	<0.001
Triglycerides in Large VLDL	5.88%	4.42%	8.39%	<0.001	<0.001
Concentration of Medium VLDL Particles	2.89%	1.70%	4.44%	<0.001	<0.001
Total Lipids in Medium VLDL	4.03%	2.66%	5.94%	<0.001	<0.001
Phospholipids in Medium VLDL	2.71%	1.74%	4.32%	<0.001	<0.001
Cholesteryl Esters in Medium VLDL	-0.57%	-1.13%	-0.22%	<0.001	<0.001
Free Cholesterol in Medium VLDL	1.72%	0.76%	3.15%	<0.001	<0.001
Triglycerides in Medium VLDL	5.91%	4.19%	8.13%	<0.001	<0.001
Concentration of Small VLDL Particles	5.50%	3.93%	7.95%	<0.001	<0.001
Total Lipids in Small VLDL	5.46%	3.90%	7.72%	<0.001	<0.001
Phospholipids in Small VLDL	3.45%	2.26%	5.12%	<0.001	<0.001
Cholesterol in Small VLDL	2.77%	1.74%	4.37%	<0.001	<0.001
Cholesteryl Esters in Small VLDL	3.17%	2.08%	4.81%	<0.001	<0.001
Free Cholesterol in Small VLDL	1.70%	0.69%	2.90%	<0.001	<0.001
Triglycerides in	6.76%	4.93%	9.94%	<0.001	<0.001

Small VLDL					
Concentration of Very Small VLDL Particles	2.42%	1.48%	3.71%	<0.001	<0.001
Total Lipids in Very Small VLDL	2.58%	1.73%	4.06%	<0.001	<0.001
Phospholipids in Very Small VLDL	3.22%	2.31%	4.86%	<0.001	<0.001
Free Cholesterol in Very Small VLDL	1.89%	1.17%	2.98%	<0.001	<0.001
Triglycerides in Very Small VLDL	6.67%	4.96%	9.04%	<0.001	<0.001
Cholesterol in IDL	-0.50%	-1.04%	-0.20%	<0.001	<0.001
Cholesteryl Esters in IDL	-0.50%	-0.99%	-0.17%	<0.001	<0.001
Free Cholesterol in IDL	-0.42%	-0.84%	-0.15%	<0.001	<0.001
Triglycerides in IDL	6.41%	4.62%	8.82%	<0.001	<0.001
Free Cholesterol in Large LDL	-0.71%	-1.30%	-0.34%	<0.001	<0.001
Triglycerides in Large LDL	7.10%	5.26%	9.87%	<0.001	<0.001
Concentration of Medium LDL Particles	1.56%	0.72%	2.93%	<0.001	<0.001
Total Lipids in Medium LDL	1.59%	0.61%	2.64%	<0.001	<0.001
Phospholipids in Medium LDL	1.32%	0.31%	2.42%	0.006666667	0.007020649
Cholesterol in Medium LDL	1.02%	0.04%	1.95%	0.046666667	0.046666667
Cholesteryl Esters in Medium LDL	1.77%	0.86%	3.17%	<0.001	<0.001
Triglycerides in Medium LDL	7.36%	5.48%	11.07%	<0.001	<0.001
Concentration of Small LDL Particles	2.02%	1.23%	3.22%	<0.001	<0.001
Total Lipids in Small LDL	1.75%	0.75%	2.96%	<0.001	<0.001
Phospholipids in Small LDL	1.54%	0.86%	2.52%	<0.001	<0.001
Cholesterol in Small LDL	0.97%	0.14%	1.89%	0.013333333	0.013678161

Cholesteryl Esters in Small LDL	1.87%	0.86%	3.24%	<0.001	<0.001
Triglycerides in Small LDL	6.89%	5.20%	9.47%	<0.001	<0.001
Concentration of Very Large HDL Particles	2.05%	1.23%	3.21%	<0.001	<0.001
Total Lipids in Very Large HDL	3.24%	2.04%	4.62%	<0.001	<0.001
Phospholipids in Very Large HDL	3.41%	2.17%	5.54%	<0.001	<0.001
Cholesterol in Very Large HDL	3.99%	2.76%	5.92%	<0.001	<0.001
Cholesteryl Esters in Very Large HDL	4.66%	3.22%	6.71%	<0.001	<0.001
Free Cholesterol in Very Large HDL	1.85%	0.98%	2.96%	<0.001	<0.001
Triglycerides in Very Large HDL	3.11%	2.18%	4.72%	<0.001	<0.001
Concentration of Large HDL Particles	4.64%	3.23%	6.92%	<0.001	<0.001
Total Lipids in Large HDL	4.31%	3.01%	6.56%	<0.001	<0.001
Phospholipids in Large HDL	3.60%	2.28%	5.31%	<0.001	<0.001
Cholesterol in Large HDL	5.81%	4.08%	9.35%	<0.001	<0.001
Cholesteryl Esters in Large HDL	6.35%	4.77%	9.23%	<0.001	<0.001
Free Cholesterol in Large HDL	4.00%	2.81%	6.00%	<0.001	<0.001
Triglycerides in Large HDL	0.76%	0.34%	1.38%	<0.001	<0.001
Phospholipids in Medium HDL	-0.43%	-0.89%	-0.15%	<0.001	<0.001
Triglycerides in Medium HDL	4.19%	3.01%	6.08%	<0.001	<0.001
Concentration of Small HDL Particles	-1.33%	-2.42%	-0.42%	0.013333333	0.013678161
Cholesterol in Small HDL	-1.82%	-2.93%	-0.82%	<0.001	<0.001
Cholesteryl Esters	-2.39%	-3.70%	-1.41%	<0.001	<0.001

in Small HDL					
Triglycerides in Small HDL	7.68%	5.89%	10.95%	<0.001	<0.001

Abbreviations: CHD, coronary artery disease; CMM, cardiometabolic multimorbidity; FDR, false discovery rate; HDL, high-density lipoprotein; IDL, intermediate-density lipoprotein; LDL, low-density lipoprotein; T2DM, type 2 diabetes mellitus; VLDL, very low-density lipoprotein.

Table S8 Sensitivity analysis of Associations of sleep pattern with the risks of CMDs and CMM.

Categories	Sensitive analysis 1 HR [95% CI]	Sensitive analysis 2 HR [95% CI]
**CMM**		
Healthy	1.00 (Reference)	1.00 (Reference)
Moderate	1.176 1.073 1.288	1.167 1.057 1.288
Poor	1.630 1.382 1.923	1.589 1.326 1.904
Per 1 decrement of healthy sleep score	1.147 1.100 1.196	1.142 1.091 1.194
**Total CMD**		
Healthy	1.00 (Reference)	1.00 (Reference)
Moderate	1.140 1.108 1.174	1.146 1.111 1.182
Poor	1.420 1.339 1.506	1.423 1.336 1.516
Per 1 decrement of healthy sleep score	1.100 1.085 1.115	1.103 1.087 1.119
**Individual CMD**		
**T2DM**		
Healthy	1.00 (Reference)	1.00 (Reference)
Moderate	1.153 1.095 1.213	1.130 1.068 1.196
Poor	1.582 1.445 1.732	1.545 1.397 1.708
Per 1 decrement of healthy sleep score	1.137 1.111 1.164	1.131 1.103 1.161
**Stroke**		
Healthy	1.00 (Reference)	1.00 (Reference)
Moderate	1.107 1.036 1.182	1.089 1.017 1.166
Poor	1.237 1.066 1.434	1.257 1.075 1.468
Per 1 decrement of healthy sleep score	1.064 1.031 1.099	1.059 1.024 1.095
**CHD**		
Healthy	1.00 (Reference)	1.00 (Reference)

Moderate	1.155 1.113 1.200	1.176 1.131 1.224
Poor	1.368 1.264 1.481	1.381 1.269 1.503
Per 1 decrement of healthy sleep score	1.095 1.075 1.114	1.104 1.084 1.125

Sensitive analysis 1: Sensitivity analysis of Associations of sleep pattern and healthy sleep score with the risks of cardiometabolic disease and cardiometabolic multimorbidity by excluding outcomes occurring within 2-year follow-up; Sensitive analysis 2: Sensitivity analysis of Associations of sleep pattern and healthy sleep score with the risks of cardiometabolic disease and cardiometabolic multimorbidity by excluding participants with low-lipid drug. Abbreviations: CHD, coronary artery disease; CMD, cardiometabolic disease; CMM, cardiometabolic multimorbidity; T2DM, type 2 diabetes mellitus.

Table S9 Associations of sleep pattern and sleep score with the risks of cardiometabolic disease and cardiometabolic multimorbidity with weighted/unweighted sleep score.

Categories	No. of cases	Incidence (1/1000)	HR1 (95% CI)	HR2 (95% CI)
**CMM**				
Healthy	783	0.80	1.00 (Reference)	1.00 (Reference)
Moderate	1739	1.19	1.201 (1.103, 1.308)	1.252 (1.128, 1.389)
Poor	218	2.13	1.653 (1.419, 1.926)	2.241 (1.775, 2.829)
Per 1 decrement of sleep score	2740	1.08	1.151 (1.108, 1.196)	1.199 (1.150, 1.250)
**Total CMD**				
Healthy	7770	8.26	1.00 (Reference)	1.00 (Reference)
Moderate	15307	11.03	1.148 (1.117, 1.180)	1.160 (1.129, 1.193)
Poor	1525	16.11	1.430 (1.352, 1.512)	1.684 (1.521, 1.865)
Per 1 decrement of sleep score	24602	10.15	1.105 (1.090, 1.119)	1.136 (1.119, 1.153)
**Individual CMD**				
**T2D**				
Healthy	2329	2.40	1.00 (Reference)	1.00 (Reference)
Moderate	5175	3.59	1.155 (1.099, 1.213)	1.250 (1.190, 1.312)
Poor	699	7.04	1.613 (1.479, 1.758)	1.786 (1.494, 2.135)
Per 1 decrement of sleep score	8203	3.26	1.143 (1.118, 1.168)	1.163 (1.138, 1.190)
**Stroke**				
Healthy	1562	1.60	1.00 (Reference)	1.00 (Reference)
Moderate	2803	1.92	1.106 (1.039, 1.177)	1.099 (1.035, 1.166)
Poor	229	2.24	1.240 (1.078, 1.427)	1.284 (0.934, 1.765)
Per 1 decrement of sleep score	4594	1.81	1.060 (1.029, 1.093)	1.083 (1.047, 1.121)
**CHD**				

Healthy	4696	4.91	1.00 (Reference)	1.00 (Reference)
Moderate	9156	6.45	1.169 (1.128, 1.211)	1.192 (1.142, 1.244)
Poor	831	8.41	1.371 (1.272, 1.477)	1.680 (1.482, 1.904)
Per 1 decrement of sleep score	14683	5.93	1.101 (1.083, 1.120)	1.134 (1.113, 1.156)

HR1 (95%CI): main analyses.

HR2 (95%CI): repeated analyses with weighted sleep score.

Abbreviations: CHD, coronary artery disease; CMD, cardiometabolic disease; CMM, cardiometabolic multimorbidity; HR, hazard ratio; T2DM, type 2 diabetes mellitus.

Table S10 Associations of sleep pattern and sleep score with the risks of cardiometabolic disease and cardiometabolic multimorbidity, adjusted/not adjusted for BMI

Categories	No. of cases	Incidence (1/1000)	HR1 (95% CI)	HR2 (95% CI)
**CMM**				
Healthy	783	0.80	1.00 (Reference)	1.00 (Reference)
Moderate	1739	1.19	1.201 (1.103, 1.308)	1.312 (1.205, 1.428)
Poor	218	2.13	1.653 (1.419, 1.926)	2.065 (1.774, 2.403)
Per 1 decrement of sleep score	2740	1.08	1.151 (1.108, 1.196)	1.227 (1.181, 1.275)
**Total CMD**				
Healthy	7770	8.26	1.00 (Reference)	1.00 (Reference)
Moderate	15307	11.03	1.148 (1.117, 1.180)	1.217 (1.184, 1.251)
Poor	1525	16.11	1.430 (1.352, 1.512)	1.650 (1.562, 1.744)
Per 1 decrement of sleep score	24602	10.15	1.105 (1.090, 1.119)	1.150 (1.136, 1.166)
**Individual CMD**				
**T2D**				
Healthy	2329	2.40	1.00 (Reference)	1.00 (Reference)
Moderate	5175	3.59	1.155 (1.099, 1.213)	1.321 (1.258, 1.388)

Poor	699	7.04	1.613 (1.479, 1.758)	2.244 2.060 2.445
Per 1 decrement of sleep score	8203	3.26	1.143 (1.118, 1.168)	1.256 1.228 1.284
**Stroke**				
Healthy	1562	1.60	1.00 (Reference)	1.00 (Reference)
Moderate	2803	1.92	1.106 (1.039, 1.177)	1.113 (1.046, 1.185)
Poor	229	2.24	1.240 (1.078, 1.427)	1.262 (1.098, 1.451)
Per 1 decrement of sleep score	4594	1.81	1.060 (1.029, 1.093)	1.066 (1.034, 1.098)
**CHD**				
Healthy	4696	4.91	1.00 (Reference)	1.00 (Reference)
Moderate	9156	6.45	1.169 (1.128, 1.211)	1.205 (1.163, 1.248)
Poor	831	8.41	1.371 (1.272, 1.477)	1.478 (1.372, 1.592)
Per 1 decrement of sleep score	14683	5.93	1.101 (1.083, 1.120)	1.125 (1.106, 1.144)

HR1 (95%CI) adjusted for age, sex, ethnicity, Townsend deprivation index, education attainment, annual household income, alcohol intake, physical activity, smoking status, BMI, hypertension, history of diabetes, and cardiovascular disease.

HR2 (95%CI) adjusted for age, sex, ethnicity, Townsend deprivation index, education attainment, annual household income, alcohol intake, physical activity, smoking status, hypertension, history of diabetes, and cardiovascular disease.

Abbreviations: CHD, coronary artery disease; CMD, cardiometabolic disease; CMM, cardiometabolic multimorbidity; HR, hazard ratio; T2DM, type 2 diabetes mellitus.
